# A composite electrodynamic mechanism to reconcile spatiotemporally resolved exciton transport in quantum dot superlattices

**DOI:** 10.1126/sciadv.adh2410

**Published:** 2023-10-20

**Authors:** Rongfeng Yuan, Trevor D. Roberts, Rafaela M. Brinn, Alexander A. Choi, Ha H. Park, Chang Yan, Justin C. Ondry, Siamak Khorasani, David J. Masiello, Ke Xu, A. Paul Alivisatos, Naomi S. Ginsberg

**Affiliations:** ^1^ Department of Chemistry, University of California Berkeley, Berkeley, CA 94720, USA.; ^2^ Department of Materials Science and Engineering, University of Washington, Seattle, WA 98195, USA.; ^3^ Department of Chemistry, University of Washington, Seattle, WA 98195, USA.; ^4^ STROBE, National Science Foundation Science and Technology Center, University of California Berkeley, Berkeley, CA 94720, USA.; ^5^ Department of Physics, University of California Berkeley, Berkeley, CA 94720, USA.; ^6^ Materials Science Division and Molecular Biophysics and Integrated Bioimaging Division, Lawrence Berkeley National Laboratory, Berkeley, CA 94720, USA.; ^7^ Kavli Energy NanoSciences Institute at Berkeley, Berkeley, CA 94720, USA.

## Abstract

Quantum dot (QD) solids are promising optoelectronic materials; further advancing their device functionality requires understanding their energy transport mechanisms. The commonly invoked near-field Förster resonance energy transfer (FRET) theory often underestimates the exciton hopping rate in QD solids, yet no consensus exists on the underlying cause. In response, we use time-resolved ultrafast stimulated emission depletion (STED) microscopy, an ultrafast transformation of STED to spatiotemporally resolve exciton diffusion in tellurium-doped cadmium selenide–core/cadmium sulfide–shell QD superlattices. We measure the concomitant time-resolved exciton energy decay due to excitons sampling a heterogeneous energetic landscape within the superlattice. The heterogeneity is quantified by single-particle emission spectroscopy. This powerful multimodal set of observables provides sufficient constraints on a kinetic Monte Carlo simulation of exciton transport to elucidate a composite transport mechanism that includes both near-field FRET and previously neglected far-field emission/reabsorption contributions. Uncovering this mechanism offers a much-needed unified framework in which to characterize transport in QD solids and additional principles for device design.

## Teaser

Multimodal measurement uncovers a multimodal mechanism for electronic energy transport in next-generation optoelectronics.

## INTRODUCTION

**Fig. 1.**
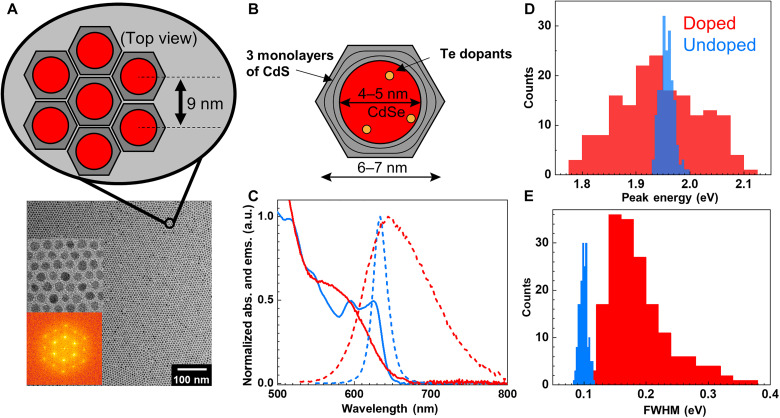
Configuration and spectral properties of Te-doped CdSe/CdS QDSLs. Te dopants in CdSe/CdS QDs broaden the absorption and emission spectra by increasing both the intrinsic linewidth and bandgap energy heterogeneity. (**A**) The hexagonal prism shape of the CdS shell promotes formation of a monolayer QD hexagonal SL with 9-nm lattice spacing, in which we investigate exciton migration dynamics, as shown in transmission electron microscopy (TEM) and the schematic cartoon. Insets include a zoomed-in region of the TEM image and Fourier transform of the TEM image. (**B**) Schematic of the internal composition of the QDs used, including 4- to 5-nm spherical CdSe shell, ~3 monolayers of CdS shell in a short, hexagonal prism shape to form a 6- to 7-nm QD size, and presence of Te dopants. (**C**) Steady-state QD absorption in colloidal suspension (solid curves) and QDSL emission spectra (dashed curves). Doped QD spectra are indicated in red; undoped QD spectra are shown in blue. a.u., arbitrary units. Single-particle emission spectroscopy was used to obtain (**D**) the emission peak energy distribution of doped and undoped QDs and (**E**) the emission spectra full width at half maximum (FWHM) of doped and undoped QDs.

Colloidal quantum dots (QDs), semiconductor nanocrystals suspended in solutions due to their surface-bound ligand molecules, are highly tunable building blocks for next generation solid-state devices, such as displays (*1*), lasers (*2*), or solar cells (*3*). QD superlattices (QDSLs) are highly ordered arrangements of QDs (Fig. 1A) that can enable bottom-up design of hierarchically organized functional materials (*4*–*6*). In general, SLs are desirable over disordered dropcast films of QDs for their high spatial order and superior transport properties. The primary photoexcited species in QDs is an exciton—an electron-hole pair that resides on a QD due to confinement effects. Exciton transport is therefore an important process for the function of QD-based optoelectronic solid devices. Understanding exciton transport mechanisms is a key step toward developing design principles that enable finer control over exciton transport. For example, QD-based solar cells benefit from high exciton diffusivity because these photogenerated excitations must travel from the site of absorption to a charge separation interface (*7*); in displays, little-to-no exciton diffusivity is desirable because exciton transport among QDs of different emitting colors or to quenching sites will cause color accuracy and low photoluminescence yield issues (*8*).

Exciton transport in QD solids has increasingly been studied via spatiotemporally resolved photoluminescence (*9*, *10*) and transient absorption (*11*, *12*) to determine the relationship between transport and microstructure and to reveal the nature, e.g., normal or anomalous diffusion, of transport. Förster resonance energy transfer (FRET) is the most commonly invoked model for exciton transport in QD solids (*9*, *13*–*15*) in which the ligand coat is long (e.g., ~2 nm) and insulating, as the center-to-center distances between QDs are therefore large. However, FRET theory often underestimates exciton diffusivity compared to experimentally derived values (*9*, *11*, *16*, *17*). Many explanations have been proposed, yet no conclusive cause has been determined. Separately, photon emission and reabsorption have been recently demonstrated to be important for exciton transport in optoelectronics. For example, repeated emission and reabsorption due to total internal reflection, i.e., waveguiding, often referred to as photon recycling, have enhanced transport in metal halide perovskite thin films and nanocrystal solids (*18*, *19*). Its potential impact in other materials, including in the well-studied colloidal II to VI semiconductor QD solids, is relatively unexplored. While both mechanisms involve coupling of donor and acceptor transition dipole moments (TDMs), the distance scalings of their respective exciton transfer rates are quite different. FRET, a near-field phenomenon, scales as *r*^−6^, while emission and reabsorption occur in the far-field, scaling as *r*^−2^. Discerning these two mechanisms with spatiotemporal microscopy alone is, however, challenging because additional constraints are needed to properly specify a model that includes them both.

The evolution of the exciton energy could generate the additional needed constraints. Both near-field and far-field mechanisms require energetic resonance between a donor and an acceptor QD. Better overlap of the donor emission spectrum and acceptor absorption spectrum leads to a higher probability of exciton transfer. Because of the presence of a Stokes shift, a small energy relaxation of an electronic excitation, the bias of transferring an excitation from a donor to a lower-energy acceptor and, thus, a corresponding decay of the average exciton energy are present in both mechanisms. This bias leads to time dependence and correlation of the exciton energy and diffusivity during transport, especially because the bandgap energies of QDs in a solid are generally distributed over a range of values, restricting the number and proximity of potential acceptors of a given donor QD’s exciton. Exciton diffusivity is therefore reflected not only in spatiotemporally resolved microscopy but also in measurements of the decay of the exciton energy as a function of time mediated by either near-field or far-field mechanisms. However, the roles of energetic resonance and distance scaling in determining the energy transfer rate through these two different mechanisms differ. The donor-acceptor distance plays a much more important role in FRET than in emission/reabsorption. Intuitively, given a specific amount of energy loss over the exciton lifetime, emission/reabsorption should generate more substantial transport because finding a resonant acceptor QD is not as challenging in a heterogeneous energy landscape as it is in FRET because there are no severe distance constraints. Thus, measuring both the exciton mean energy and diffusivity as a function of time (*9*) should allow the relative contributions of the two mechanisms to be quantified and distinguished.

Here, we indeed elucidate mixed-mechanism excitonic energy transport by combining time-dependent exciton energy and diffusivity measurements in a heterogeneous QDSL energy landscape, leveraging a multimodal background-free fluorescence approach. We find that FRET and emission/reabsorption together explain exciton transport behavior in highly ordered Te-doped CdSe/CdS core-shell QDSL monolayers, with a smaller far-field contribution providing a substantial increase to the overall transport. To do so, we first quantitatively characterize the heterogeneous energetic landscape of CdSe:Te/CdS QDSL monolayers by extracting inhomogeneous and intrinsic spectral components of QDs by single-particle emission spectroscopy (*20*, *21*). We next track the time-dependent decay of the mean exciton energy by time-resolved emission spectra (TRES) after QDSL photoexcitation. Last, we measure exciton transport by monitoring the spatiotemporal expansion of an exciton population after a local photoexcitation in QDSL monolayers with time-resolved ultrafast stimulated emission depletion (TRUSTED) microscopy (*22*) and use all three types of experimental results to constrain parameters in a kinetic Monte Carlo (KMC) simulation. Our findings reveal that often overlooked far-field dipole-dipole coupling is an important energy transfer mechanism to include for colloidal QD solids. Furthermore, the relatively high reported exciton migration length demonstrates the possibility of using doped QDs to decouple energetics from QD size in optoelectronic devices.

## RESULTS

To discern FRET and emission/reabsorption in QDSLs, we focus on 5% Te-doped CdSe (core)/CdS (shell) QDs (diameter, 6 to 7 nm) with mixed oleic acid and oleylamine ligands whose shape resembles a short hexagonal prism, such as the nut of a nut and bolt (Fig. 1, A and B). This nanocrystal shape allows for us to achieve highly ordered domains of self-assembled nanocrystals where the nanocrystals have well defined interparticle spacing, crystallographic orientation, and minimal structural disorder (*23*). These readily hexagonally pack into an ordered SL. Sample preparation and characterization are described in sections S1 and S2. The primary effect of Te doping is to broaden the QD emission spectra (*24*) as shown in Fig. 1C (red curve), in contrast to the undoped control (blue curve). Single-particle emission spectroscopy of the 5% Te-doped CdSe/CdS QDs in comparison to their undoped CdSe/CdS control QDs shows that both the distribution of single QD emission peak energies (Fig. 1D) and spectral widths (Fig. 1E) increase substantially with doping (see section S1 and fig. S1), suggesting that the increased emission bandwidth observed in bulk solution in Fig. 1C is due to broadening on both the single QD and ensemble levels. After forming a QDSL monolayer (section S2 and figs. S2 to S6), the emission peak redshifts relative to the solution emission.

**Fig. 2.**
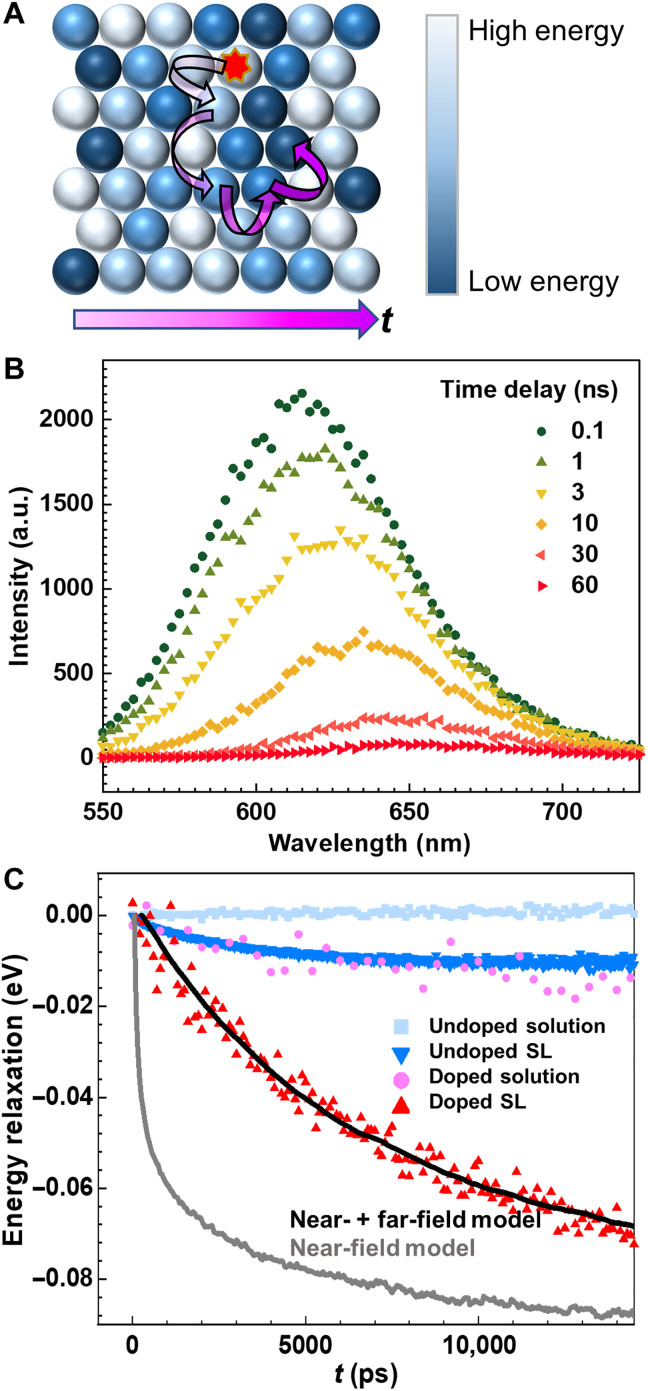
Exciton migration dynamics tracked energetically by TRES. (**A**) Cartoon of exciton (red star) hopping from a high-energy site (pale blue shades) to a low energy site (dark blue shades). Time is indicated with increasingly deep magenta. (**B**) Time-dependent photoluminescence spectra at select time delays after photoexcitation of a CdSe:Te/CdS QDSL. The emission peak energy shows an increasing redshift with increasing time delay. (**C**) TRES of doped QD solution (pink circles) and SLs (red triangles) track the decay of mean exciton energy. Undoped solution (light blue squares) and SL (dark blue triangles) counterparts are also shown for reference. Simulated energy decay from spatial-spectral dynamics of near-field (gray) and near-field plus far-field (black) models is also shown.

**Fig. 3.**
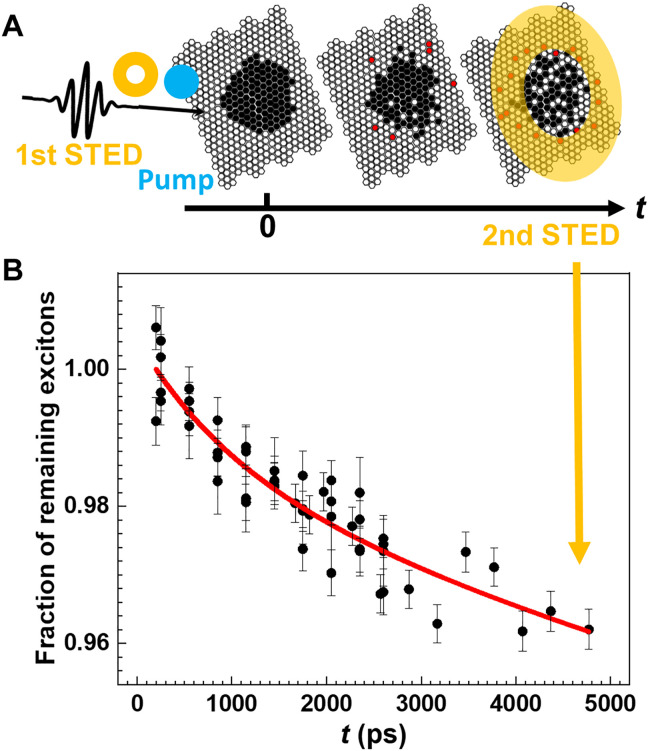
Exciton migration dynamics tracked spatially via TRUSTED. (**A**) Schematic diagram of a TRUSTED protocol. Black circles are excitons on the QDSL lattice (otherwise open circles) excited by the pump laser pulse and not quenched by the first STED laser pulse. Red circles correspond to excitons that migrate into the (yellow) annular area illuminated by the STED laser pulse intensity that are thus quenched upon the time-delayed arrival of a second STED pulse. (**B**) Measured TRUSTED fraction of remaining excitons (black points) decays as a function of time delay between first and second STED pulse arrivals is fit (red) using an exponentially decaying diffusivity model. Decreasing fraction corresponds to exciton diffusion.

To track the time-dependent evolution of the average exciton energy and migration (schematics in Fig. 2A), both of which are needed to determine the relative contribution of near- and far-field dipole-dipole coupling in the QDSL monolayer, we use TRES and TRUSTED, respectively. TRES of the CdSe:Te/CdS QDSL are shown in Fig. 2B, and the peak energy emission redshift is evident as the time delay increases. The associated time resolved mean energy relaxation is shown in Fig. 2C for the doped QDs in solution (pink) and monolayer QDSL (red) along with the corresponding controls for undoped QDs (light and dark blue, respectively). The energy loss plotted corresponds to the difference between the average emission energy at each recorded time delay following ~100-ps photoexcitation at 470 nm and the peak emission energy at zero time delay. QDs have been demonstrated to be amenable to stimulated emission depletion (STED) super-resolution fluorescence microscopy (*25*, *26*). Here, we use its ultrafast adaptation, TRUSTED. To characterize exciton migration in the QDSL monolayer, we report the fraction of excitons remaining at a given time delay after a subdiffraction population is prepared within the monolayer in a TRUSTED experiment (Fig. 3, A and B). The initially prepared population of excitons consists of those generated by a few-picosecond diffraction-limited 550-nm pump pulse (Fig. 3A, blue) that is not quenched by a nearly coincident 100-ps 740-nm annular “STED” pulse (Fig. 3A, yellow). The remaining excitons at a given time delay correspond to those that are not quenched by an identical annular STED pulse at that time delay [see Materials and Methods and (*22*) for further details]. The net result is that the data in Fig. 3B) may be fitted to a simple model of the TRUSTED protocol (*22*) (Fig. 3B, red curve) to determine migration parameters, such as diffusivity. At present, TRUSTED time delays of up to ~5 ns can be recorded with ~100-ps time resolution, making TRES’s much longer time delay window highly complementary. The TRES and TRUSTED data, along with single-particle and bulk emission spectroscopy, are used to constrain a KMC simulation of exciton transport that tracks both energy and location of excitons schematically represented in Fig. 2A. Below, we first describe the results obtained from the two experimental methods and then describe how these are analyzed in combination with the simulations.

We focus first on the TRES results in Fig. 2C. For both solution-phase QDs and QDSLs, we focus our comparison of the decay and characteristic decay time within the first 15 ns of the full window of 150 ns measured (figs. S7 and S8), when the signal-to-noise ratio is highest. As shown in Fig. 2C, a more pronounced dynamic redshift in the QDSL [9.8 meV for the undoped (dark blue) and 80 meV for the doped (red)] is observed from TRES compared to from QD solutions (~0 for the undoped in light blue and 15 meV for the doped in pink). In addition, the Te-doped samples have a greater magnitude of redshift over the exciton lifetime than their undoped counterparts. The enhanced dynamic redshift occurring in the QDSLs relative to the solution-phase QDs immediately following excitation indicates the existence of nonequilibrium exciton transport. That is, the average exciton energy initially decreases as a function of time. This observation means that the additional redshift in the SL state is not a property of isolated QDs but a result from the interaction among QDs. The large redshift in the doped QDSL suggests that its energetic landscape is very heterogeneous (Fig. 1C), in qualitative agreement with single-particle emission spectroscopy results (Fig. 1D), in addition to the breadth of the constituent QD energies also obtained from single-particle measurement (Fig. 1E). Similarly, the undoped QDSL exhibited a smaller amplitude of energy relaxation (Fig. 2C, dark blue), in accordance with the narrower energy distribution in Fig. 1D (blue).

We use TRUSTED to spatially resolve exciton migration within the CdSe:Te/CdS QDSL monolayers, whose broadened and redshifted emission relative to the absorption onset is ideal for STED microscopy. The undoped CdSe/CdS QDSL system has too small of a Stokes shift to be amenable to STED microscopy or TRUSTED. We fit our data in Fig. 3B with a time-dependent diffusivity model (red curve). The initial diffusivity is (4.6 ± 2.5) × 10^−3^ cm^2^/s, and a roughly fourfold decrease in the exciton diffusivity occurs within the 5-ns measurement window. In a TRUSTED dataset, the horizontal axis is the time delay between the first and second STED pulses. The vertical axis corresponds to the fraction of remaining excitons, calculated as the ratio of the photoluminescence with and without the second STED pulse, and then normalized to its initial value to construct the “fraction of remaining excitations” in the detection volume. A decaying signal means that excitons move outward between the time delay of the two STED pulses, and the slope of the decay depends on the rate at which excitons travel. In the simplest model, excitons are assumed to undergo a random walk, and the aggregated behavior of these excitons is similar to Brownian motion. A time-dependent diffusivity fit to the TRUSTED data is warranted, given that TRES reveal that the mean exciton energy decreases with time. As an exciton proceeds to sample lower energy sites, the number of acceptor sites concomitantly decreases, which would lead to a time-dependent reduction in the energy transfer rate. By approximating this decay with an exponentially decaying exciton diffusivity model using *D*(*t*) = *D*_o_ exp(−*kt*) + *D*_c_, the fit diffusivity at time *t* = 0 is *D*_o_ = (4.6 ± 2.5) × 10^−3^ cm^2^/s, the decay rate *k* = (0.0008 ± 0.0010) ps^−1^, and the long-time diffusivity *D*_c_ = (1.5 ± 1.2) × 10^−3^ cm^2^/s. Because of the limited signal-to-noise ratio, the error bars on these cited diffusivity parameters are somewhat substantial. Nevertheless, the uncertainty in the characteristic extent of exciton transport within the TRUSTED measurement window, $\sqrt{\int D(t)\cdot dt}$ is small: 35 ± 6 nm. (The extrapolated characteristic extent of exciton transport over the QD lifetime, typically referred to as *L*_D_, is ~47 nm.) Various control experiments to ensure that the decay of the TRUSTED signal originates from exciton transport are provided in section S4 and figs. S9 and S10. In addition, measurements of 2 and 7% CdSe:Te/CdS QDSL monolayers can be found in section S4 and figs. S11 to S14. They yield exciton diffusivities of the same order of magnitude as the 5% Te-doped sample. (The synthesis of these nanocrystals occurred in a different batch from the 5% doped ones. To avoid convolving the associated potential differences in QD properties with the differences in doping concentrations, we refrain from making meaningful comparisons between the 5% batch and the 2 and 7% batch.)

To best relate the above energy relaxation and energy migration dynamics, we first characterized the 5% CdSe:Te/CdS QD’s heterogeneous energy landscape with single-particle emission spectroscopy. As shown in Fig. 1D, compared to undoped CdSe/CdS QDs, the 5% CdSe:Te/CdS emission peak distribution is much wider, indicating a more heterogeneous distribution of QD bandgap energies. The heterogeneity is most likely a result of variability in Te doping among individual nanocrystals, such as the relative number and/or location of dopants within a given QD, as opposed to size variability within the ensemble. Franzl *et al.* (*27*) demonstrated two distinct subpopulations of Te dopants in CdSe/ZnS QDs probed via single-particle emission spectroscopy. One subpopulation had one to a few Te dopants and a similar linewidth to undoped particles, and another subpopulation had many Te dopants and much broader spectra. Our single-particle emission results suggest that at 5% doping, our QDs likely only show the “many dopants” regime proposed by Franzl *et al.* (*27*), as we do not observe a distinct population of “few dopants.” Given the differences in our synthesis procedure such as higher temperature, it would not be unexpected that our spectroscopy differs from the much earlier work of Franzl *et al.*, likely resulting from higher and more homogeneous Te incorporation within the CdSe core.

Having established the nature of the energy landscape of the QDSL monolayers, we can not only assert that TRES in QD monolayers reveal the decay of mean exciton energy in QDSL monolayers caused by exciton transport (Fig. 2, A and C), but that the rates of mean energy decay and exciton transport are proportional to one another. Since the mean energy decay rate *k*_Δ*E*_ in the undoped QDSL (Fig. 2C, dark blue) is larger than that of the doped QDSL (Fig. 2C, red), exciton transport in the undoped QDSL appears to be faster than the doped case. We deduce that the extra redshift in the SL state is not a property of the isolated QDs (Fig. 2C, pink) but a result of the interaction among QDs. A spectral shift absent in QD solution but present in QD solids is often taken as evidence for energy transport (*9*, *16*, *28*) facilitated by the QD proximity in solid films. During this energy transport, excitons, on average, sample progressively lower energy QDs as they explore the spatioenergetic landscape (Fig. 2A). The TRES decay amplitude is therefore a measure of energy heterogeneity, and the decay rate is proportional to the exciton transport rate. Furthermore, the single-particle emission spectroscopy and the TRES measurement of the QD energy heterogeneity support the picture of exciton hopping occurring from high-to-low energy sites on a heterogeneous energetic landscape. A single-exponential fit to the energetic relaxation of the form Δ*E* = Δ*E*_∞_[1 − exp(−*k*_Δ*E*_*t*)] is performed, where ${\text{Δ}E}_{\infty }=-\frac{{\sigma_{\mathrm{ih}}}^{2}}{k_{B}T}$, σ_ih_ is the width of the inhomogeneous distribution of site energies, and *k*_B_*T* is the thermal energy at room temperature (26 meV) (*29*). On the basis of this fit, *k*_Δ*E*_ = 0.045 ns^−1^ and σ_ih_ = 56 meV for the doped QDSL, a bit smaller than the result from single-particle emission spectroscopy, which is 74 meV after converting from full width at half maximum in Fig. 1C. Using the ~±20 meV spread in the measured TRES energy loss at long times (fig. S8) as a bound for uncertainty of σ_ih_, this value agrees well with the single-particle emission spectroscopy.

## DISCUSSION

**Fig. 4.**
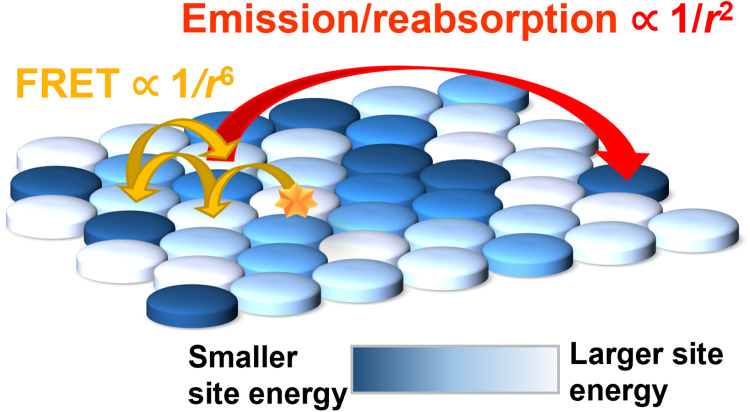
Depiction of composite energy transport mechanism. Schematic illustrating the inclusion of both near-field FRET hops and the occasional far-field emission/reabsorption over a heterogeneous energy landscape made up of sites with smaller (dark blue) and larger (white) exciton energies.

We have presented two measurements that inform on exciton transport in the doped QDSLs—TRES, monitoring the rate of the mean energy change, and TRUSTED direct spatiotemporal measurements of the expanding exciton spatial distribution. Although FRET is the most commonly invoked mechanism to describe energy transfer between QDs with long alkyl ligands (*9*, *16*, *30*, *31*), we find that it is inadequate to justify the experimentally observed exciton diffusivity and to reconcile the TRES and TRUSTED results: FRET predicts an exciton diffusivity 100 to 1000 times smaller than the diffusivity reported by TRUSTED (section S5); furthermore, an energy relaxation rate consistent with the TRUSTED observation, according to a generously parametrized FRET model, is much faster than the rate obtained by TRES. The canonical *R*_o_ parameter, the distance at which energy transfer is 50% efficient, would have to be unphysically high to recapitulate the observed diffusivity. This discrepancy has been reported previously by others, and additional considerations investigated include the degree to which a point-dipole approximation is valid, the potential contribution of higher-order dipole-quadrupole interactions, the potential for more optimal dipole-dipole orientation in a solid assembly, and the potential for a larger absorption cross section in films than what is estimated solution (*16*, *17*, *32*). To illustrate the incompatibility of the TRUSTED and TRES results within a FRET framework, we show in Fig. 2C that if *R*_o_ is adjusted to match the exciton diffusivity extracted by TRUSTED, then it predicts a much faster decay of the mean exciton energy than TRES reports (Fig. 2C, gray curve). We find that our two measurements can, however, be reconciled by including contributions from higher-order terms in the dipole-dipole coupling expansion elaborated below from which FRET retains only the steep *r*^−6^ near-field term (Fig. 2C, black curve). With *r*^−6^ dependence alone, exciton transfer is largely limited to one to two shells of nearest-neighbor QDs, and the spectral overlap (requirement of exciton energy resonance) between QDs has less effect on the transfer rate than the distance between QDs. However, alleviating the distance penalty for the exciton transfer by introducing higher-order (far-field emission/reabsorption) terms allows hops well beyond the nearest neighbors that enjoy better energy resonance, just as sketched in Fig. 4. As we show below, even very few of these hops result in a much higher effective diffusivity, where the decay in mean exciton energy remains slow enough to match TRES.

To arrive at this conclusion, we developed a KMC simulation of energy transport by dipole-dipole coupling of QD TDMs, in which the intermediate (*r*^−4^) and far-field (*r*^−2^) terms of the dipole-dipole interaction expansion can be included. As explored by Andrews (*33*) and Juzeliūnas and Andrews (*34*), the energy transfer rate between donor TDM and acceptor TDM can most generally be written as

$$k_{\mathrm{DA}}=(k_{\mathrm{near}}+k_{\mathrm{intermed}}+k_{\mathrm{far}})\exp(-\alpha r)$$ (1)

where 1/α is the attenuation length of photons absorbed when traveling inside the medium of donors and acceptors, set here to 400 nm, based on measurement (*35*), and $k_{\mathrm{near}}=\frac{1}{\tau_{D}}\;\frac{R_{o}^{6}}{r^{6}}$ as already described. The terms *k*_intermed_ and *k*_far_ also both depend on $\frac{1}{\tau_{D}}$, the reciprocal of the exciton lifetime, but scale with *r*^−4^ and *r*^−2^, respectively, with *r* being the dipole-dipole distance. Whereas the standard FRET *r*^−6^ term dominates in the near field in which the donor-acceptor distance is much smaller than the reduced wavelength λ/2π, the far-field *r*^−2^ term dominates when *r* is much greater than λ/2π, and the intermediate *r*^−4^ term is important when the donor-acceptor separation is comparable to λ/2π.

A major contribution to *R*_0_^6^ is the overlap integral between acceptor absorption and donor emission spectra, which is weighted by a function of the optical frequency ω^−4^ (or, λ^4^): $\int \sigma_{A}(\omega )\bar{F_{D}(\omega )}\omega^{-4}d\omega$. [Here, σ_A_(ω) is the wavelength-dependent absorptivity of the acceptor chromophore species, and $\bar{F_{D}(\omega )}$ is the fluorescence emission spectrum normalized by its area; see sections S5 and S6.] The corresponding spectral overlap integral for the far-field contribution is simply $\int \sigma_{A}(\omega )\bar{F_{D}(\omega )}d\omega$ without any spectral weighting, and the intermediate term is weighted by ω^−2^. Relative to the near-field term, the scalings of these weightings effectively enhance the higher-order terms 4- and 17-fold, respectively. In our KMC simulation, we therefore embodied Eq. 1 by introducing correlated unitless coefficients for the two higher-order terms

$$k_{\mathrm{DA}}=\frac{1}{\tau_{D}}\;\frac{R_{o}^{6}}{r^{6}}\left[1+c_{\mathrm{intermed}}{\left(\frac{2\pi }{\lambda }\right)}^{2}r^{2}+c_{\mathrm{far}}{\left(\frac{2\pi }{\lambda }\right)}^{4}r^{4}\right]\exp(-\alpha r)$$ (2)

in which *c*_intermed_ ≅ √*c*_far_ to reflect these differences. While we expect boundary conditions associated with the field and medium geometry to also play a role (see below), these coefficients introduce a relative importance between terms that should be sufficient to reflect Eq. 1 in the KMC simulation.

When *c*_far_ ~ 27, it is possible to use the same model parameters to simultaneously fit both the TRES decay (Fig. 2C, black) and the exciton diffusivity extracted via the TRUSTED measurements in Fig. 3B, and the value returned for *R*_0_ is a very reasonable 7.6 nm. Full details of the simulation can be found in section S6, including an exploration of the role of the different terms in the expansion that demonstrates more explicitly how neither near- nor far-field term alone is able to simultaneously recapitulate the TRUSTED energy transport and TRES energy decay (fig. S15). This exploration further supports that a small amount of emission/reabsorption (far-field interaction) is consistent with our data and suggests that a similar interpretation may explain other spatiotemporal transport studies in QD solids and potentially other systems. Our hypothesis is that both near-field and far-field couplings occur. The near-field coupling predominantly supports exciton hopping to nearest neighbors of the originally excited QD (see section S6) and, on its own, would require *R*_0_ = 15 nm, beyond acceptable FRET radii. The modest 6% fraction of hops associated with the far-field coupling (section S6) enables an overall measured exciton migration length of ~35 nm within the first 5 ns. Far-field effects have been demonstrated in many other systems, including perovskite thin films (*36*). Whereas thin films are sufficiently thick to support traditional waveguiding and enhance the far-field contribution via photon recycling, no traditional waveguide modes can be supported in materials as thin as QDSL monolayers, and more complex mechanisms are needed to explain the relative enhancement of far-field over near-field coupling contributions.

The electrodynamic treatment of the donor-acceptor transfer rate in Eq. 1 or 2 accounts for a substantial portion of *c*_far_ ~ 27. Namely, the combination of the ratio of different overlap integrals of the near- and far-field terms and different orientational factors (see section S6) accounts for all but a factor of ~5. There are multiple additional potential contributors to this stronger relative far-field contribution that satisfies the simultaneous constraints imposed by the TRUSTED and TRES experimental results. First, Eqs. 1 and 2 assume isotropic orientational distributions of TDMs, which do not necessarily accurately reflect the weighting of donor TDM orientations in the QDSL monolayer. One primary contribution to reweighting the TDM distribution is that the dielectric boundary conditions can strongly affect the radiative rate when the donor and acceptor are confined to a narrow plane of dielectric constant greater than the surrounding media, as is presently the case. Consider the QDSL monolayer as a nanoconfined cavity, i.e., a 9-nm-thick slab whose dielectric constant exceeds those of its surroundings. Emission from TDMs oriented parallel to the monolayer plane should be entirely suppressed in the present limit that the cavity thickness is much lesser than the emission wavelength. Nevertheless, TDMs perpendicular to the plane retain nonzero emission amplitude (*37*, *38*) and can furthermore be enhanced because of the Purcell effect—an alteration of the radiative rate due to proximity to a dielectric object (*39*). Typical dipole radiation patterns of TDMs oriented normal to the plane favor emission in the plane via a sin^2^θ dependence, an effect that is not explicitly accounted for in Eq. 2 but that can indeed be encapsulated in *c*_far_, and which is distinct from the orientational contributions to $R_{o}^{6}$. Furthermore, the emission direction probability can be substantially modified on the basis of the specific cavity thickness and the dielectric mismatch between the cavity and the surroundings, with strong in-plane enhancements (*40*, *41*). Although a full quantum electrodynamics treatment with the appropriate boundary conditions for this system is beyond the scope of the current work, the combination of radiative enhancement and in-plane directional bias provides another explanation for the apparent relative strength of the far-field contribution to the donor-acceptor transfer rate in Eq. 2. We solidify this claim through simulations of the enhancement of far-field to near-field strengths in the plane of an explicit lattice of TDMs in the presence of a dielectric medium in section S7 and fig. S16. Last, a number of recent reports describe additional plasmonic, polaritonic, or evanescent waveguiding transport mechanisms (*42*–*44*), which could additionally contribute to the stronger far-field contribution consistent with our observations. Regardless, from our data presented herein, we find that only by including interactions via far-field dipole-dipole coupling do we obtain a model capable of reconciling the two independent measurements of energy transport, TRES and TRUSTED, in CdSe:Te/CdS QDSL monolayers, and we presented multiple possible explanations for the model parameters thus obtained, the most substantial of which fall directly out of the electrodynamic equations.

This work provides a more general treatment of dipole-dipole coupling–mediated energy transfer in electronically coupled systems that go beyond near-field interactions. Prompted by explaining our exciton transport observation in colloidal QD solids, subject to importantly included spectroscopic constraints, for a heterogeneous energetic landscape, we have shown the value of tracking and correlating the energetic relaxation and spatial expansion of excitons in the system under study to distinguish different energy transport pathways. Different materials that support exciton transport may differ in the relative balance of near-field (FRET) and far-field (emission/reabsorption) energy transfer events exhibited. To enhance long-range exciton transport, the two mechanisms call for different optimization strategies. For example, to enhance the far-field mechanism, which can offer strong advantages in increasing the efficacy of an optoelectronic device in which the energy transport material must direct as many excitons as possible to a specific location to transduce their energy into useful work, decreasing the concentration of chromophores could be helpful, so that photons emitted in-plane can travel further before reabsorption and to suppress FRET. In addition, designing materials with high dielectric constants or having the exciton host material sandwiched by high dielectric constant materials could enhance far-field coupling through Purcell-like effects. Alternatively, when near-field coupling easily dominates the far-field coupling due to the distance scaling, excitons can be better spatially contained, which is helpful in display applications. By decreasing Purcell-like effects through index matching of materials surrounding the active layer, far-field coupling could be deliberately reduced. Last, to optimize long-range near-field/FRET-like transport, achieving a more homogeneous site energetic landscape is clearly important to prevent exciton trapping at a low energy site, but in the absence of this homogeneity, far-field contributions can compensate.

We noted earlier that other spatiotemporal measurements of exciton transport in QD solids have generated results that suggest transport in excess of what is predicted by FRET theory. We propose that incorporating the possibility of higher-order terms in the dipole-dipole coupling of donor and acceptor TDMs could reconcile not only our findings but also those of others that have remained at odds with FRET. For example, Akselrod *et al. (**9*), who measured both spatial and spectral diffusion via time-resolved photoluminescence, found a characteristic migration length of 32 nm, which would require *R*_o_ to be 11 nm, although the expected *R*_o_ based on steady-state properties is around 5.5 nm; Mork *et al.* (*16*) found that the experimental *R*_o_ between a donor and an acceptor QD is ~9 nm, while the estimation based on the classic formula is ~5 nm and explore various possible explanations without arriving at a specific consensus. Applying our model to a specific example from Akselrod *et al.*, we find that their measured migration length would include a modest amount of emission/reabsorption with *c*_far_ ~ 35 very comparable to our own and a decrease in *R*_o_ to a more acceptable value of 5 nm (section S8). Further investigating related patterns from other measurements may provide a much-needed unified framework in which to characterize QD solids and with which to leverage different device design principles.

In conclusion, we present a comprehensive study of the exciton transport mechanism in a CdSe:Te/CdSe QDSL monolayer that reconciles larger-than-expected exciton diffusivities. To do so, we measured the spatiotemporal dynamics of exciton diffusivity via TRUSTED and the accompanying spectrotemporal mean energy relaxation dynamics via TRES. Combining these with explicitly measured QD energy heterogeneity extracted from single-particle measurements, we deduced that the apparent diffusivity must be an average of near- and far-field transport. Such a conclusion would not be possible without all three measurements, since these leverage spectroscopic, in addition to spatial and temporal observables. Together, these different datasets provide sufficient constraints to supply to a KMC simulation model of exciton transport to elucidate the composite transport mechanism, which would not have been possible with only a subset of the techniques used. With this model, we infer that far-field interactions should even more markedly enhance exciton transport when the energy inhomogeneity is lower (see section S9 and table S2). This powerful multimodal approach is generally applicable and can be readily applied not only to other QD solids but to other optoelectronic materials. The recognition of far-field contributions to exciton transport by including them in a transport model also promises to better explain spatiotemporal transport measurements more broadly, resolving a long-standing paradox presented by the use of FRET-exclusive models. Incorporating this model directly into optoelectronics device design will furthermore assist in generating more optimal functions.

## MATERIALS AND METHODS

### Sample preparation

Te-doped CdSe/CdS QD with oleic acids and oleylamine ligands were synthesized with further details in the Supplementary Materials. They self-assemble into a QDSL at liquid-air interfaces and are then transferred to a glass substrate. The sample is then encapsulated in a nitrogen atmosphere with ultraviolet-cured epoxy before optical characterization.

### Time-resolved ultrafast stimulated emission depletion

TRUSTED (*22*) is used in a home-built confocal microscope with a 63× 1.4 numerical aperture Plan Apo Leica objective (HC PL APO 63×/1.40 oil CS2, Leica Material #11506350). The excitation and depletion laser pulse trains at 200 kHz were derived from third-harmonic and second-harmonic noncollinear optical parametric amplifiers (Light Conversion), respectively, pumped by a 10-W Light Conversion PHAROS regeneratively amplified laser system with a fundamental wavelength of 1030 nm. The 25-fJ excitation pulse was centered at 550 nm with 20-nm bandwidth, and the two 125-pJ depletion pulses were centered at 740 nm with a bandwidth set to 16 nm. Both the pump and depletion (STED) pulses were fiber-coupled into single-mode polarization-maintaining fibers to produce high-quality Gaussian modes. A vortex phase mask (RPC Photonics VPP-1a) was then used to generate the annular depletion pulse beam mode. The pulses were then directed through a quarter waveplate positioned to circularly polarize the depletion pulses. During the experiment, the sample is rastered with a PI Nano scanning piezoelectric stage (P-545.3C7) in steps of 15 μm over a 60-μm by 60-μm area. Data from the resulting 25 spatial locations are averaged to improve the signal-to-noise ratio. Epifluorescence is collected between 687.5 and 712.5 nm through dichroic mirrors and emission filters (three 700/25 filter from Edmund Optics) and is focused onto a fast-gated SPAD detector with a 200-ps rise time (A. Tosi, SPAD lab, Politecnico di Milano; PicoQuant) controlled by a Picosecond Delayer (MPD) that is triggered just after the arrival of the second depletion pulse to eliminate fluorescence occurring before the definition of the detection volume. We phase-lock the detection data stream to the timing of an optical chopper (Newport 3501) placed in the excitation pulse line, so that we may separately determine the photon count rates during the “excitation on” and “excitation off” chopper phases for multiple cycles. The count rates obtained during these open and closed phases of the chopper are each corrected for the classic pile-up effect with a simple Poisson correction factor (*45*) before we take the difference of the two to isolate the count rate that is attributed to the modulated excitation pulse only. The second STED pulse is separately modulated with a shutter so that data collected when this pulse is blocked can be used as a reference and control. The signal versus delay time obtained when this second depletion pulse is unblocked is divided by the signal versus delay time observed when it is blocked. The resulting data are then normalized to the extrapolated value of this ratio at zero delay time to calculate the fraction of remaining excitations in the detection volume as a function of the delay time.

### Time-resolved emission spectra

The sample was pumped by a 470-nm (Picoquant diode laser) laser with ~100-ps temporal resolution. Photoluminescence spectra from 550 to 750 nm were collected in a Picoquant FluoTime 300 Fluorometer at normal incidence with wavelength step size of 2.5 nm for the CdSe:Te/CdS QDSL sample. Luminesced photons were collected by a photomultiplier tube detector, and the factory-set wavelength-dependent response of the photomultiplier tube was used as a calibration curve to correct the individual spectra. The peak emission wavelength was extracted by fitting the peak in the time-dependent photoluminescence spectra to a Gaussian function.

### Single-particle emission spectroscopy

Solutions of QDs in hexanes were diluted to single-particle concentrations of ∼100 pM, deposited on a cleaned microscope slide, and quickly encapsulated with a coverslip. Single-particle emission spectra were recorded under a wide-field scheme, as described previously (*20*, *21*). Briefly, the sample was mounted on a setup based on a Nikon Ti-E inverted fluorescence microscope and imaged in the wide field with an oil-immersion objective lens (CFI Plan Apochromat λ 100×, 1.45 numerical aperture) under 488-nm illumination. Emission was split into two channels for the concurrent recording of single-particle images and spectra, with the latter enabled by inserting a dispersive prism into the light path.

### KMC simulation

Simulations of incoherent exciton hopping trajectories were performed with discrete hops on a 5760-nm by 5760-nm two-dimensional hexagonal lattice with periodic boundary conditions with a lattice constant of 9 nm. For each trajectory, site energies were randomly assigned in accordance with the experimentally measured inhomogeneous broadening width, and trajectories were initiated at a random site of the two-dimensional lattice. Hopping rates between pairs of sites were then calculated from the site energies, the site-to-site separation, the intrinsic spectral width of the sites, and the intrinsic Stokes shift, in a dipole-dipole coupling model (see the Supplementary Materials for details). Approximately 20,000 trajectories were averaged for each set of parameters, and the resulting mean diffusivities were used to estimate the diffusion extent within TRUSTED experimental windows and within the exciton lifetime.

## Supplementary Materials

### This PDF file includes:

Supplementary Text Figs. S1 to S16 Tables S1 and S2 References
