# Supplementary material for: A composite electrodynamic mechanism to reconcile spatiotemporally resolved exciton transport in quantum dot superlattices
Source: Sci Adv. 2023 Oct 20;9(42):eadh2410. doi: 10.1126/sciadv.adh2410 (PMC10588942; doi:10.1126/sciadv.adh2410)
Supplement: Supplementary file 1 — Supplementary Text Figs. S1 to S16 Tables S1 and S2 References [file sciadv.adh2410_sm.pdf]

Science Advances
AAAS

Supplementary Materials for

A composite electrodynamic mechanism to reconcile spatiotemporally resolved exciton transport in quantum dot superlattices

Rongfeng Yuan *et al.*

Corresponding author: Naomi S. Ginsberg, nsginsberg@berkeley.edu

Sci. Adv. **9**, eadh2410 (2023)
DOI: 10.1126/sciadv.adh2410

This PDF file includes:

Supplementary Text
Figs. S1 to S16
Tables S1 and S2
References

1. **Sample preparation**:

Substrate. Glass coverslips were sonicated for 10 minutes in successive baths of acetone, isopropyl alcohol, 2% Hellmanex solution in NANOpure deionized water, and 2X rinses in NANOpure deionized water, and immediately dried with filtered nitrogen flow. Coverslips were further cleaned with oxygen plasma in a reactive ion etch chamber.

Chemicals. Cadmium oxide (CdO, ≥99.99%, Sigma Aldrich), octadecylphosphonic acid (ODPA, 99%, PCI Synthesis), trioctylphosphine oxide (TOPO, 99%, Sigma Aldrich), trioctylphosphine (TOP, 99%, Strem), selenium powder (Se, 99.999%, Sigma Aldrich), tellurium powder (Te, 99.8%, Sigma Aldrich), oleic acid (OA, 90% tech grade, Sigma Aldrich), oleylamine (OAm, 70% tech grade, Sigma Aldrich), 1-octadecene (ODE, 90% tech grade, Sigma Aldrich), n, n-dimethylformamide (DMF, anhydrous 99%, Sigma-Aldrich), toluene (anhydrous 99.5%, Sigma Aldrich), acetone (anhydrous, 99.5%, Sigma Aldrich), hexanes (mixture of isomers, anhydrous 95%, Sigma Aldrich), n-octane (98%, reagent grade, Sigma Aldrich). 0.2 M $Cd(oleate)_2$ in ODE precursor was prepared using a previously reported procedure(*46*). Briefly, appropriate amounts of CdO, OA, and ODE were degassed for about two hours under vacuum at 110 °C until all gases and water had evolved. The reaction was then heated to 240 °C under argon for ~ 30 minutes until a clear, slightly yellow, solution forms. The flask was then cooled to 110 °C and degassed a second time for an additional 1-2 hours to remove additional water. The solution was stored in an argon-filled glovebox.

Wurtzite CdSe:Te{5%} Nanocrystals. Te-doped CdSe core nanocrystals were synthesized by modifying a similar procedure for undoped CdSe nanocrystals(*47*). Briefly, 120 mg CdO, 560 mg ODPA, 6 g TOPO were degassed under vacuum in a 50 mL round-bottom flask at 150 ºC for 1 hour. The mixture was then heated to 320 ºC under argon gas and maintained at 320 ºC until solution turned clear indicating formation of a complex. Upon completion of complex formation, 3 g of TOP was injected in the solution and then the temperature was raised to 360 ºC. A solution of the appropriate ratio of Se and Te totaling 1.5 mmol in 0.72 g TOP was swiftly injected when temperature approached 360 ºC. Upon injection, CdSe nanocrystals were formed and were allowed to grow for 90 seconds to produce large, uniform cores. Solution was then rapidly cooled with compressed air to 120 ºC before injecting 10 mL of anhydrous toluene and transferring to an inert atmosphere. The nanocrystals were cleaned in an inert environment with successive precipitation and redispersion using acetone and hexane respectively and were stored in 3ml of hexane. Concentration were determined by a previously reported empirical formula(*48*) and size was determined via TEM image analysis.

Hexagonal CdSe:Te{5%}/CdS core/shell Nanoplates: A hexagonal CdS shell typically 3 monolayers thick was grown around the Te-doped CdSe nanocrystals(*49*). The anisotropic shell growth was grown by first degassing under vacuum 3 mL of ODE, 3 mL of OAm and 100 nmol of CdSe cores in a 25 mL round-bottom flask at 110 ºC for 30 mins. The solution was then heated to 310 ºC under Ar. When the temperature reached 240 ºC, injections of Cd and S precursor solutions in ODE started at a rate of 3 ml/hr using a syringe pump. The Cd precursor solution contained ~4 mL of 0.20 mM $Cd(OA)_2$ and the S precursor contained ~25 mg of S powder in ~3 g of TOP. Upon completion of the precursor injections, the reaction was maintained at 310 ºC for 10 minutes, then cooled rapidly using compressed air to room temperature. Successive precipitation and redispersed using acetone and hexane respectively

were done to clean the nanocrystals capped with OA and OAm before they were stored in 2 mL hexane in an inert atmosphere.

QDs without Te dopants were also prepared. To match the peak emission wavelength of the Te-doped QDs, we increased the undoped CdSe core size and kept the same 3 monolayer CdS shell.

Self-assembly of Nanocrystal Superlattice: Formation of nanocrystal superlattice was performed using a previously published self-assembly method(*50*). Monolayer nanocrystal superlattices were formed at the liquid-air interface at room temperature. The nanocrystal stock solution was diluted in octane to a proper concentration to obtain monolayer coverage. Approximately, 50 uL of diluted nanocrystal solution was drop-casted on top of 1mL of DMF in a 1cm^2 square Teflon well. The well was covered with a glass cover slip to slow solvent evaporation rate overnight. The self-assembled superlattice was scooped onto a plasma cleaned glass cover slip for optical measurements or a Cu 400 mesh standard carbon TEM grid for structural characterization. For optical measurements, the substrate is glass coverslips.

2. Sample characterization

Single QD photoluminescence spectra. Steady state photoluminescence spectra of 170 individual doped QDs and 182 individual undoped QDs were recorded according to the procedure described in the Materials and Methods section. Below, three examples of 5% Te doped individual QD photoluminescence spectra (orange, blue, and red curves) and the average of 170 such spectra (dashed red curve) are plotted. The statistics of the peak energy and FWHM distributions in Figure 1d and e are extracted from these 170 individual PL spectra.

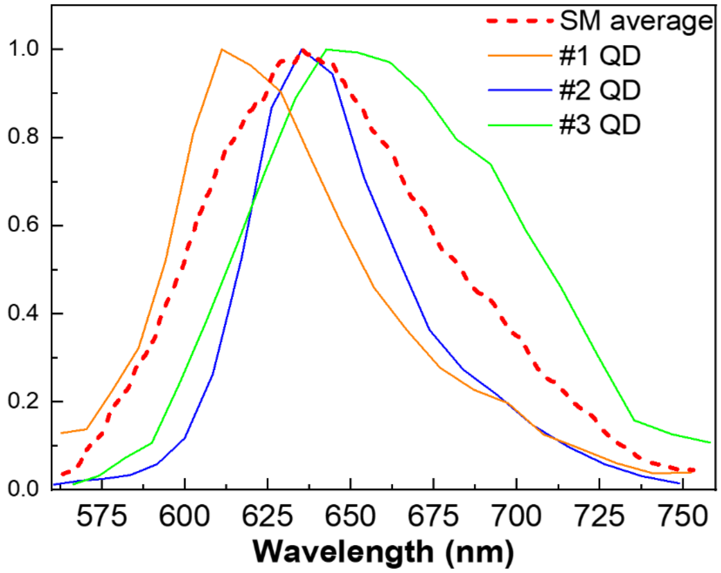

Figure S1. Example of three individual 5% Te doped QDs PL spectra and the average of 170 such spectra.

<u>Absorption and emission spectra of undoped and 5% Te doped QD</u>. Steady state absorption and emission spectra of sample in hexane solution verify the successful incorporation of the Te dopants within the CdSe matrix, shown in **Figure S2**. Absorption spectra were collected with a Shimadzu UV-3600 double beam spectrometer operating with 1 mm slits. Photoluminescence (PL) spectra were obtained with a 470nm excitation laser and Picoquant FluoTime 300 Fluorometer. The absorption spectra of undoped CdSe/CdS and doped CdSe:Te/CdS systems are shown as solid curves and their PL emission are shown as dashed curves. The absorption spectra are scaled to aid in comparison, and the emission spectra are normalized to their peak emission intensity.

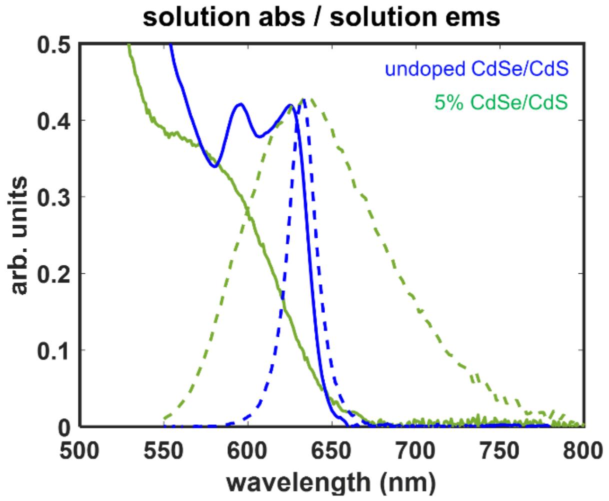

Figure S2: **Steady state absorption and emission spectra of just undoped and 5% doped solution.**

Both the undoped QD solution's and the 5% Te doped QD solution's PL emission peaks at ~632 nm. The width of the doped emission spectra is substantially wider. The FWHM of the undoped CdSe/CdS QDs in solution is ~65 meV, and for the doped profiles, ~286 meV.

When measured in the superlattice solid-state, the PL emission of both systems redshift (shown in **Fig. 1** in the main text). The undoped QDSL PL emission peaks at ~634 nm, and the doped emission profiles peak at 646 nm. The FWHM of the undoped QDSL emission profile is ~72 meV, and for the doped profiles, ~284 meV.

For the 5% CdSe:Te/CdS QD solution, the absorption onset is smeared and renders assigning a first exciton peak less obvious. Here we used a crude way to estimate the Stokes shift. The absorption lineshape was fit with a double Gaussian function, one to capture the shoulder (sloping baseline) and one to capture the absorption onset. This yields a 586 nm absorption peak and a 160 meV Stokes shift, which is in line with a previous estimate(*24*).

X-ray Diffraction: X-ray diffraction was measured of nanocrystal sample drop-casted on a silicon substrate. XRD was collected on a Bruker Phaser D2 diffractometer with a Cu kα source operated at 30kV and 10 mA with a 160 SSD detector. Diffraction measurement was done from 20° to 60° 2Θ with increments of 0.01° with an integration time of 3 s/step.

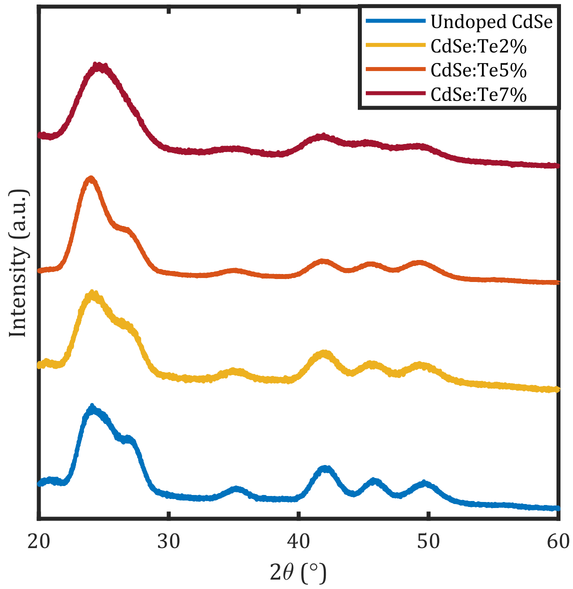

Figure S3: **X-ray diffraction pattern of Te-doped CdSe/CdS core shell quantum dots.**

From the powder X-ray diffraction patterns, we observe that the incorporation of Te atoms in the CdSe matrix does not substantially affect the CdSe crystal structure of the nanocrystal. CdSe and CdTe quantum dots can be synthesized to have either a wurtzite(wz-) or a zinc blende(zb-) crystal structure. The quantum dots used in the work have been prepared by modifying a known high-temperature synthesis of wz-CdSe(*47*). It has been reported that when synthesized at high temperatures, the zb-CdTe crystal structure is favored over wz- CdTe(*51*). All wurtzite peaks are present for the Te-doped and undoped CdSe quantum dots. As we increase the amount of Te dopant in the CdSe host matrix, we observe a broadening of the diffraction peaks due to minor change in the lattice parameter due to Te having a larger atomic radius than Se. For the CdSe:Te{7%} sample which has the most Te dopants, the broadening of the peaks lead to a merging of the 101 and 002 peaks, the presence of the other wz-CdSe peaks (102,110,103,112) show that the Te dopant at these dopant levels does not substantially change the crystal structure of the wz-CdSe nanocrystals. We also do not observe any signature zb-CdTe XRD peaks in the XRD measurements. Based on these measurements, we can conclude that the Te is likely incorporated into the CdSe matrix as a dopant as opposed to the formation of a CdSe:CdTe alloy.

<u>STEM-EDX Characterization and Analysis</u>: To investigate whether the synthesis strategy yielded the expected Te doping percentage in QDs, we conducted scanning transmission electron microscopy – energy dispersive X-ray (STEM-EDX) experiments. Here, each element (Cd, S, Se, Te) can be quantified by its corresponding X-ray spectral features. Within experimental error, STEM-EDX confirmed that the Te dopant percentage, (6.2 ± 2.3) % is the same as our expectation of 5% from the QD synthesis procedure.

Elemental composition analysis of nanocrystals was performed at the National Center for Electron Microscopy, Molecular Foundry, Lawrence Berkeley National Laboratory. Samples were drop-casted on ultrathin 400 mesh carbon Cu TEM grids and left under vacuum to remove any excess solvent and oxygen plasma treated. High-angle annular dark field HAADF-STEM and STEM-EDS maps was collected on the FEI TitanX 60-300 microscope using a FEI low-background double-tilt holder. HAADF-STEM was performed at 300 kV with a beam convergence semiangle of 10 mrad using a Gatan HAADF detector. STEM-EDS mapping was performed at 300 kV with a screen current of ~2 nA using four windowless silicon drift detectors with a total solid angle of 0.7 steradians and 140 eV energy resolution. STEM-EDS maps were collected using the Bruker Esprit software for ~5-10 minutes utilizing drift correction. Quantification of the elemental composition of each sample was done using the Bruker Esprit software using the Cliff–Lorimer method for each element: Cd K-series, Se K-series, S K-series, Te L-series.

<u>TEM Characterization and Image Analysis for Verification of superlattice formation</u>: Transmission Electron Microscopy (TEM) and High Resolution TEM (HRTEM) were used to characterize nanocrystal superlattices using a FEI Tecnai T20 S-TWIN TEM operating at 200 kV with a LaB_6 filament. TEM images were collected using a Gatan Rio 16IS camera with full 4k by 4k resolution using the drift correction feature. Sizing distribution curves were generated from TEM images of > 4000 nanoparticles using a custom written MATLAB script in which the details have been previously reported(*52*). Image analysis and Fourier transform of image was performed on ImageJ software.

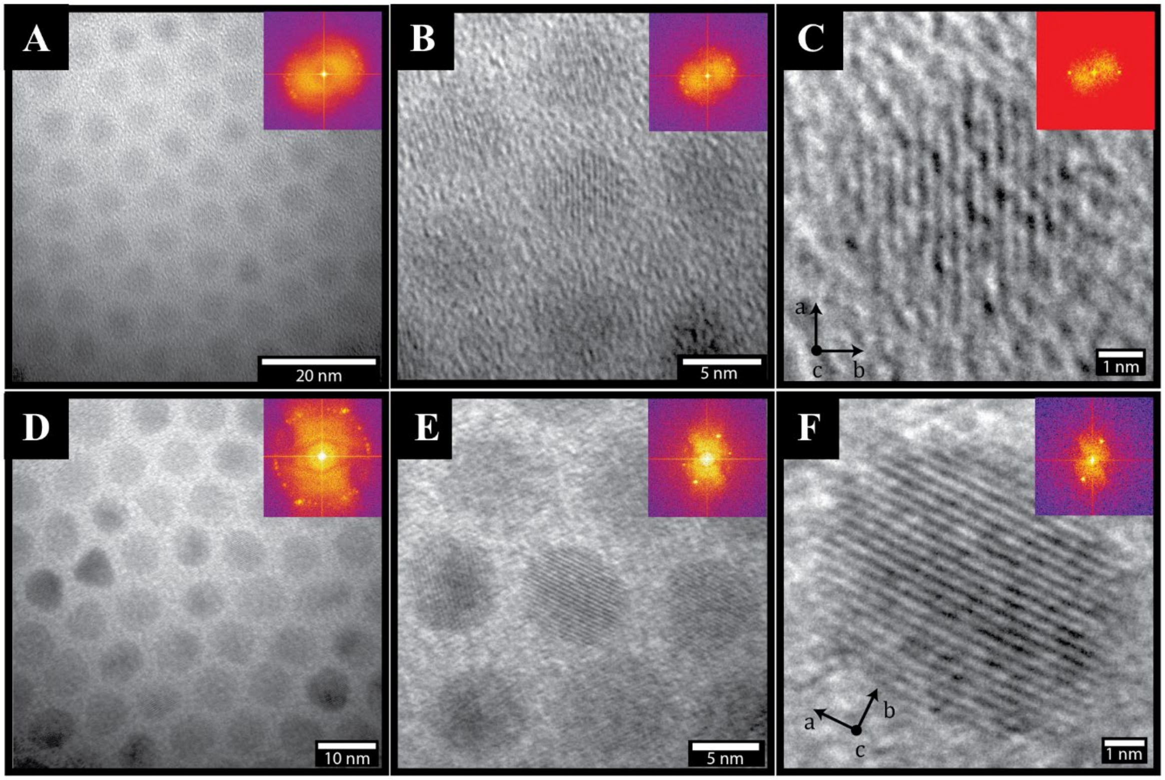

Figure S4. **HRTEM was performed on the QD superlattices to confirm the nanocrystal shape, ordering and crystallographic alignment.** For the CdSe:Te/3MLCdS samples studied in the main text we measured these highly spatially ordered superlattices (A). Based on the fast Fourier transform (FFT) inset of the image we also observe crystallographic ordering since there are discrete points in the FFT instead of rings that are often observed in disordered systems. By zooming into a smaller section of the image (B), we better observe the crystallographic alignment of these QDs as the discrete points are localized in a specific region showing that there is interparticle crystallographic alignment. The image of one CdSe:Te/3MLCdS QD (C) shows only two points in the FFT that match the regions in the FFT of the wider view images. From these images we observe that the QDs have both good spatial ordering and crystallographic ordering with the *c*-axis of the QD being perpendicular to the substrate and the a-axis mutually aligned in-plane. We performed these same analysis for the CdSe:Te/6MLCdS QDs and observed a similar trend (Fig D-F). Since the CdSe: Te/6MLCdS QDs are larger than the CdSe:Te/3MLCdS, the HRTEM images have better resolution and contrast, making it easier to see the QD edges and understand the nanocrystal shape. From the HRTEM of the CdSe:Te/6MLCdS sample (Fig D-F), we can see that these QDs have a hexagonal prism shape as discussed in the main text as opposed to an isotropic circular shape.

<u>Tapping mode AFM imaging for Verification of superlattice formation</u>: AFM images were acquired using an Asylum MFP-3D with TAP 150 AL silicon AFM probes from Ted Pella, Inc. Scans were performed with 31.0 nm per pixel resolution at a 0.5 Hz scan rate with 0.73 V driving amplitude.

AFM height mapping is useful in determining whether the large area superlattice structures we investigate are monolayers, bilayers, or higher order thicknesses. **Figure S5** is a representative AFM height map of a piece of superlattice film. The central, relatively featureless surface is approximately 6-7 nm in height and agrees well with the estimated 5 nm height of our CdSe:Te/CdS QDs (excluding the ligand layer). The additional lighter contrast features adorning the periphery of the superlattice fragment represent smaller subregions of bilayers, with an approximate height of 12-13 nm. The lightest contrast features are larger scale structures, possibly thicker disordered aggregates of QD material.

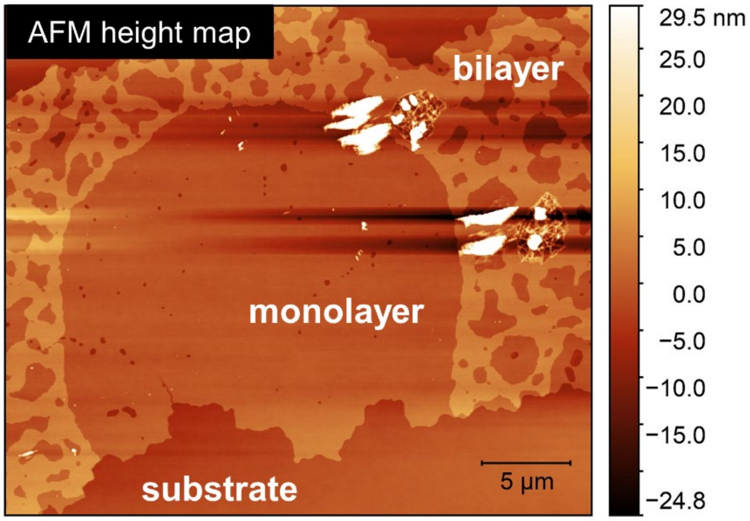

Figure S5: **AFM height image of a CdSe:Te/CdS superlattice fragment.** Labeled in the image are regions of monolayer and bilayer superlattice formation.

Brightfield optical image of QDSL monolayer and bilayer

Brightfield images were obtained by illuminating the sample with a while light LED and collecting the transmission with a ThorLabs CMOS camera. **Figure S6** shows an example image of a region of predominantly CdSe:Te/CdS monolayer superlattice (left) and a region of predominantly CdSe:Te/CdS superlattice bilayers (right). In both cases, owing to the 10s of nm thickness, we slightly defocus the image in order to more easily see the superlattice fragments. For TRUSTED experiments, we only examined monolayer locations in the whole sample.

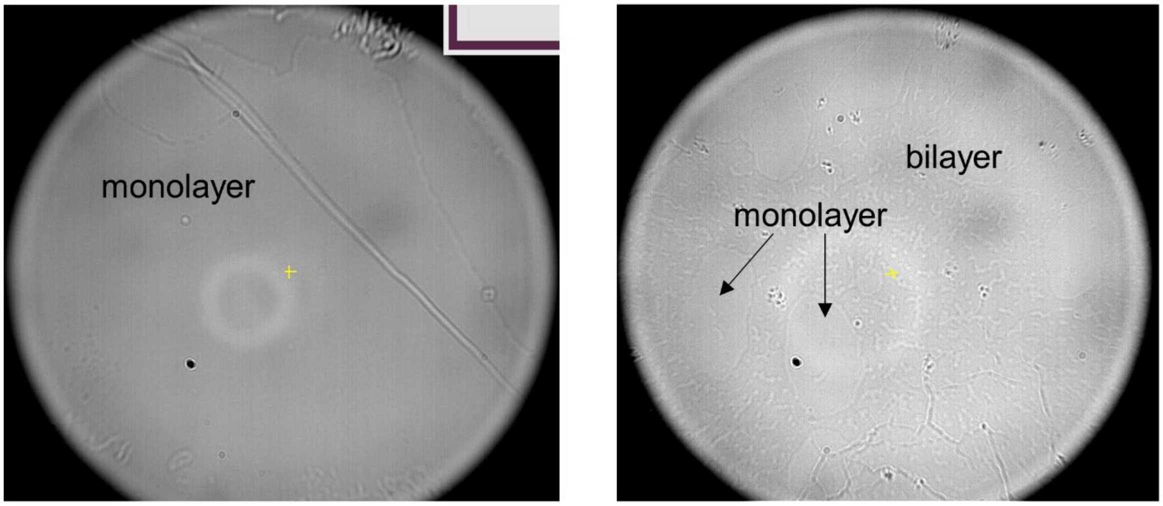

Figure S6: **Brightfield images of CdSe:Te/CdS fragments.**

3. TRES data processing and fitting procedure

Figure S7 shows the time-dependent normalized PL spectra of the doped QDSL. The factory-set photomultiplier tube (PMT) calibration curve is also plotted in forest green. The PMT has a lower sensitivity to redder photons, but the signal-to-noise ratio of TRES is large enough that we did not observe a sloping baseline in the time-dependent PL spectra. Thus, it is unlikely that the correction factor would create an artificial redshift in TRES.

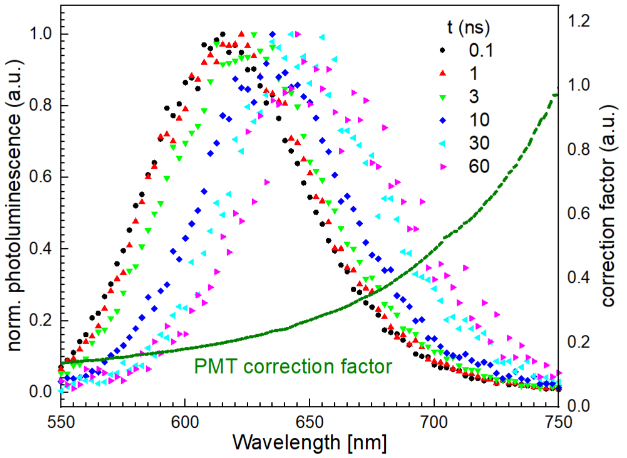

Figure S7. The time-dependent PL spectra of the doped QDSL normalized to the same peak height at the same time delays. The forest green dashed curve corresponds to the factory-set calibration curve of the PMT response.

According to Bässler(*29*), if we assume the density of site energies follows a Gaussian distribution of states, then from time-resolved emission traces one can estimate the inhomogeneous broadening components from the following expression, provided the energy relaxation in the system follows Boltzmann statistics and is within a "hopping" regime:

$$\Delta E = \Delta E_{\infty}(1 - e^{-k_{\Delta E}t})$$

Eq. S1

where $k_{\Delta E}$ is the rate constant corresponding to the rate of energy change in the system and ΔE_{∞} represents the equilibration energy, which can be related to the inhomogeneous broadening via:

$$\Delta E_{\infty} = \frac{-\sigma_{\mathrm{ih}}^2}{k_{\mathrm{B}}T}$$

Eq. S2

where σ_{ih} is the inhomogeneous broadening component of the spectral linewidth. Bässler demonstrates that **Equation S2** is a result of assuming an excitation reaches an "equilibrium" amongst lattice sites within its lifetime. The overall decrease in energy as reported by TRES is ~ 120 meV; thus, σ_{ih} is 56 meV. Note that the above treatment is only strictly valid if we assume the exciton transfer rate has an energy dependence that follows a Boltzmann distribution. This is unlikely the case for the QDSL in the strict sense. **Figure S8** shows the single exponential fit of

the time-dependent energy loss due to exciton transport. The fact that the fit is not good hints the limitation on this model, but this model still provides a sense of the relative disorder in the energetic landscape.

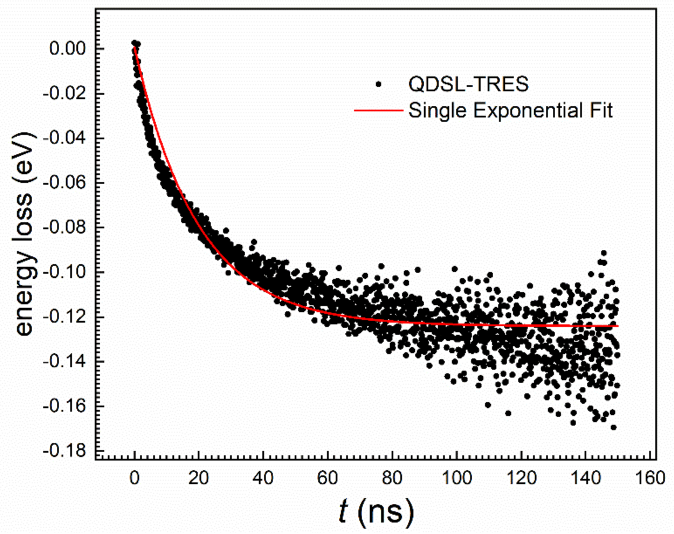

Figure S8. **Decay of mean exciton energy of a Te doped CdSe/CdS QDSL measured by time-resolved emission spectroscopy (TRES).**

4. Spatiotemporal measurement of exciton transport

STED resolution determination

One large source of uncertainty in assigning a diffusivity from TRUSTED datasets, here, is our measurement of the initial FWHM of the exciton population resulting after the action of the pump and first STED pulse. According to our resolution determination measurement, the FWHM of our initial exciton distribution is ~220 nm, where the fitting model uncertainty (weighted by the uncertainty in the data) bounds this estimate between 215 and 230 nm. See **Table S1** for diffusivity estimates using these initial FWHM estimates.

The TRUSTED fitting scheme(*22*) used to extract a diffusivity requires as an input parameter the FWHM of the exciton distribution after the action of STED1. We determined this value by imaging a sub-diffraction limited sized fragment of a 5% CdSe:Te/CdS superlattice at a series of different STED powers. **Figure S9** presents a plot of the FWHM vs STED power (measured at the sample plane), where the FWHM values are the result of fitting a line-cut of the imaged fragment using a Gaussian. The STED power we employ in our TRUSTED measurements is 25 μW at the sample plane (at 200 kHz repetition rate and ∼120 ps pulse duration). We fit the data using **Equation S3** below (red curve) and generate a 2σ error in the fit by considering both the variance in the fitting as well as the uncertainty in the data, which is the uncertainty of the linecut fits used to extract the FWHM from the imaged QDSL fragment (red shaded region).

$$FWHM_{eff} \approx \frac{FWHM_c}{\sqrt{1 + \frac{I_{STED}}{I_{sat}}}}$$

Eq. S3

According to **Figure S9**, at a 25 μW STED power we assume our resolution to be 220 nm, with an upper and lower bound of 230 and 215 nm, respectively. Fitting the TRUSTED datasets with these upper and lower bounds of the FWHM estimate, with both a constant diffusivity and time-dependent diffusivity fit, yields the results presented in **Table S1**. **Table S1** demonstrates that in all cases, the diffusivity extracted from our TRUSTED measurement is on the order of 10^{-3} cm^2/s. With regards to the time-dependent diffusivity model, we note that the time-constant of the decay is nearly identical across our three initial FWHM guesses, and the ratio of (D_o + D_c) to D_c ranges between 3.3 and 3.9. As D_c is the hypothetical diffusivity at equilibrium (i.e., as time approaches infinity), the diffusivity can conceivably decay by a factor between 3.3 and 3.9, provided an exciton lives long enough to reach equilibrium.

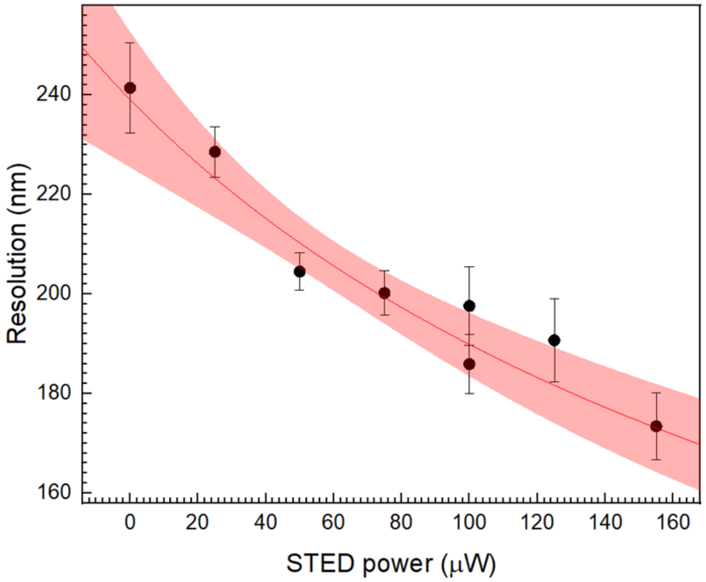

Figure S9: **Resolution vs STED power of an isolated 5% CdSe:Te/CdS QDSL fragment.** The red curve is the result of a fit to **Eq S3**, and the pink shaded region represents the 2σ range of the error of the fit weighted by the uncertainty in the data.

FWHM(nm)	D_o (x10^{-3} cm^2/s)	D_c (x10^{-3} cm^2/s)	k_d (ps^{-1})	model
215	4.0 ± 2.4	1.4 ± 1.0	0.0008 ± 0.0010	$D_o e^{-k_d t} + D_c$
220	4.4 ± 2.7	1.7 ± 1.2	0.0008 ± 0.0011	$D_o e^{-k_d t} + D_c$
230	5.5 ± 3.8	2.4 ± 1.5	0.0009 ± 0.0012	$D_o e^{-k_d t} + D_c$
215	2.3 ± 0.2	-	-	D_o
220	2.7 ± 0.3	-	-	D_o
230	3.7 ± 0.3	-	-	D_o

Table S1. TRUSTED fitting results with upper and lower uncertainty of initial FWHM.

TRUSTED control experiments

To substantiate our confidence in the TRUSTED measurement, a few key control measurements were required to rule out potential non-diffusion related contributions to the TRUSTED observable.

Exciton-exciton annihilation or Auger-Meitner recombination

Exciton density-dependent contributions to the non-radiative decay of the exciton population, such as exciton-exciton annihilation or Auger-Meitner recombination, could be mistaken for migration due to relative changes in the spatial profile leading to more pronounced overlap with the STED pulse. Provided the timescales associated with such a change to the exciton profile are within our observation window (e.g., 200 to 4800 ps) this could lead to a time-dependent change in the quenching action of the second STED pulse. To address this, we collected the PL from 25 different spatial locations on a 5% CdSe:Te/CdS monolayer at several pump powers. We plot the average of the collected PL counts from the different spatial locations as a function of pump power measured at the sample in **Figure S10**. We fit a linear trend to the lowest 5 powers and extrapolate across the entire range of pump powers measured. At powers higher than 5 nW, we note that the average PL counts begin to deviate substantially from the expected trend, which is a characteristic sign of Auger recombination or exciton-exciton annihilation.

TRUSTED measurements were conducted at 2 nW and 5 nW powers, which correspond to excitation densities of ~3.1×10^{-5} excitations/nm^3 and ~7.7×10^{-5} excitations/nm^3, respectively, indicated as arrows in **Figure S10.** The estimated diffusivities are $(2.5 \pm 0.7) \times 10^{-3}$ cm^2/s and $(1.7 \pm 0.3) \times 10^{-3}$ cm^2/s, respectively, and they are within error of one another, suggesting we are operating within a linear excitation regime.

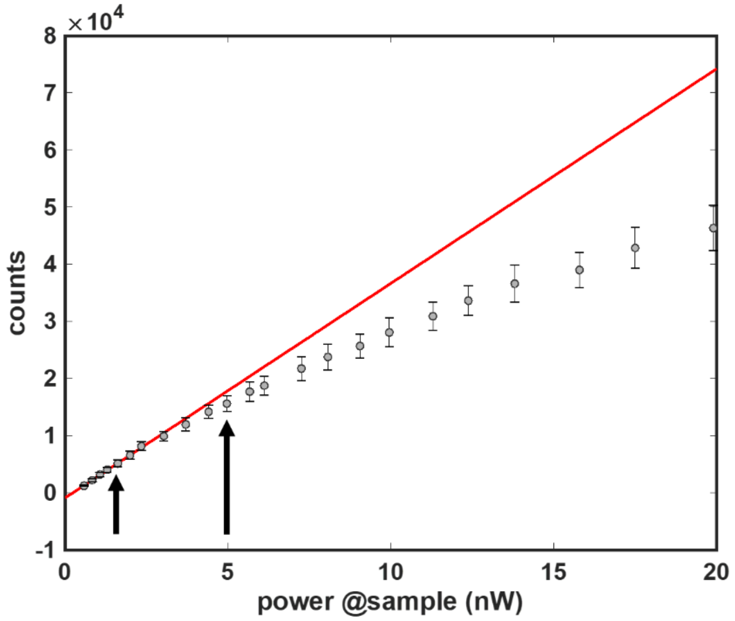

Figure S10. PL counts vs. pump power measured at the sample plane. The red curve is a linear fit determine by the lowest 5 power data points. The black arrows indicate 2 and 5 nW excitation, which are the two powers used in TRUSTED.

Exciton transport in 2% and 7% Te doped CdSe/CdS core-shell quantum dot superlattices

We characterized the exciton transport properties of QDSL monolayers of 2% and 7% Te doping levels as well. These two QDs share the same CdSe core size and CdS shell thickness as the 5% Te doped CdSe:Te/CdS QDs. We do want to note that the 2% and 7% samples are from a different batch of nanocrystals, precluding making quantitative comparisons between their time-resolved emission traces as well as TRUSTED datasets and those of the 5% samples.

First, we measured the QD solution absorption as well as QD solution and superlattice emission spectra of the 2% and 7% Te doped QD show in **Figure S11** below. For reference, they were plotted together with the undoped and 5% Te doped QD sample presented in the main text.

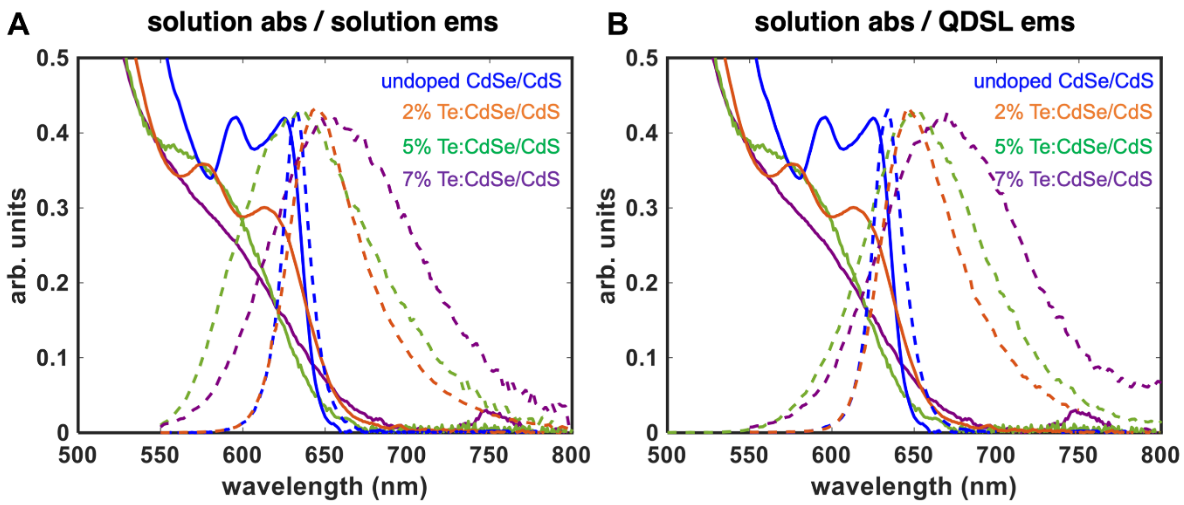

Figure S11: Steady state absorption and emission spectra of undoped and 2, 5, and 7 % Te-doped CdSe/CdS QDs. (A) Absorption and emission spectra of all 4 systems, collected in hexanes solution. (B) The same solution absorption spectra as (A), but the emission spectra are collected from the superlattices of all 4 systems.

Using TRES (**Figure S12**), we tracked the decay of mean exciton energy and found that similar to 5% Te doping, a larger dynamic redshift is present when the QDs are arranged in superlattice compared to the QDs suspended in solution. Like **Figure 2** in the main text, this suggests that there is energy transport in the QDSLs made from 2% and 7% Te doped CdSe/CdS QDs.

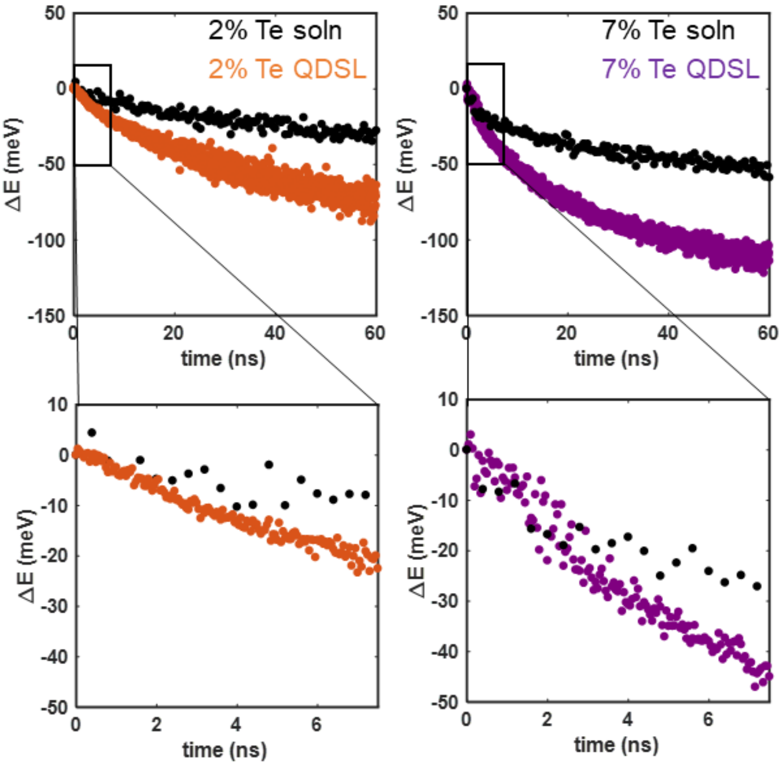

Figure S12: **TRES of 2, 7% Te doping of CdSe/CdS QDs in solution (black data) and in superlattice (colored data).**

Finally, using TRUSTED, we measured exciton diffusivities in the 2 and 7% Te-doped QDSL monolayers. The 2% and 7% data are presented in **Figure S13**. The TRUSTED data for each sample are shown, assuming a constant diffusivity model, and we obtain diffusivities of $(1.3\pm0.2)\times10^{-3}$ cm^2/s and $(1.3\pm0.5)\times10^{-3}$ cm^2/s for the 2% and 7% doped samples, respectively. We did not attempt a fit to the data with a time-dependent diffusivity model given the relatively small change in the normalized detection volume fluorescence. The relatively larger error in the 7% data is a consequence of the lower PL quantum yield of the 7% QDSL monolayers. As mentioned above, we refrain from making a direct comparison between the 5% dataset and the 2% and 7% data given we suspect batch-to-batch variability in the nanocrystal preparation.

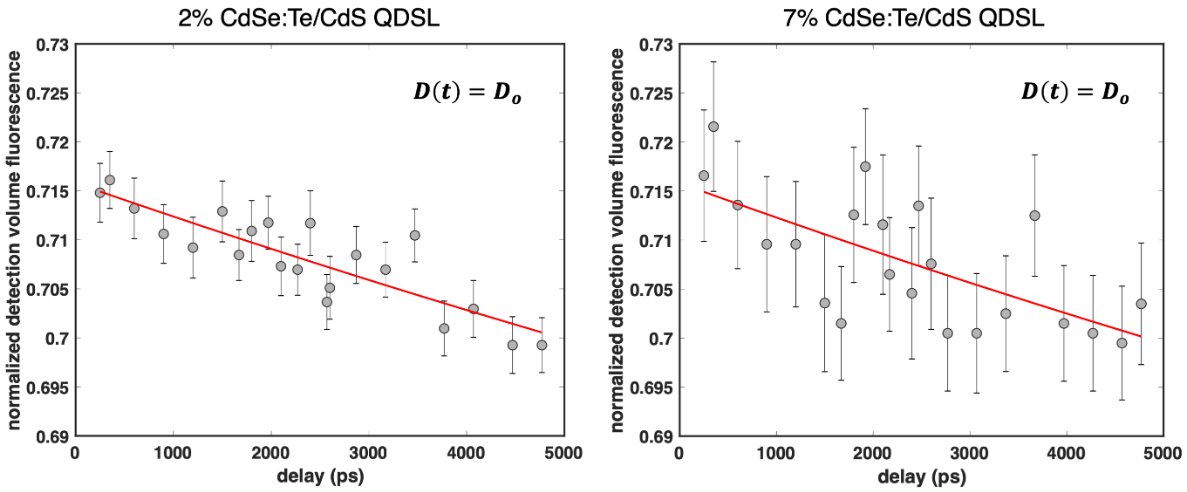

Figure S13: **TRUSTED measurements of CdSe:Te/CdS superlattice monolayers for the 2% (left) and 7% (right) Te-doping.**

One might anticipate the TRUSTED data to follow a trend as a function of the Te doping level, as is seen for the steady-state spectra and TRES measurements. It is important to note, however, that an increasing magnitude of energy relaxation in TRES does not necessarily imply faster or slower exciton transport. In fact, the energy relaxation rate is similar among the 2%, 5% and 7% doped CdSe:Te/CdS QDSL samples, especially when compared to the relaxation rate of the undoped sample as is shown in **Figure S14**.

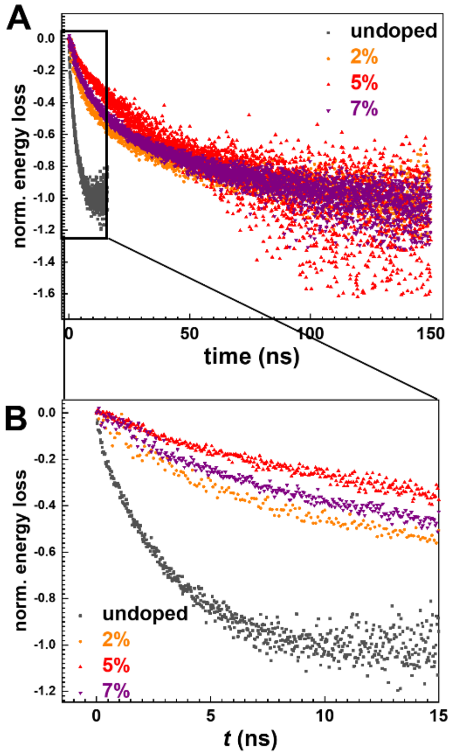

Figure S14. **Time-resolved emission of variously doped QDSL samples.** (A) Time-resolved emission data for the undoped (black), 2% doped (orange), 5% doped (red), and 7% doped (purple) QDSL samples are normalized to [0, -1]. Data extending beyond 20 ns for the undoped QDSL sample are omitted, as the lifetime of the undoped QDSL is substantially shorter than the doped QDSLs. (B) The same data are presented as in (A) but with the range limited to 15 ns.

5. FRET model for determining exciton diffusivities

Revisiting the equations describing the rate of energy transfer in a FRET framework, the rate constant is:

$$k_{FRET} = \frac{1}{\tau_D}\left(\frac{R_o}{r}\right)^6$$

Eq. S4

where τ_D is the radiative lifetime of the donor chromophore, r is the distance between the centers of the point transition dipoles used to approximate the spatial extent of the electronic transition densities corresponding to the ground-to-excited state transitions, and R_o is the "FRET radius", which is the distance at which the energy transfer rate is 50% efficient. R_o packages up many terms, and is defined as:

$$R_o^6 = \frac{9\log(10)\,\Phi_D\kappa^2}{128\pi^5\eta^4}\int \sigma_A(\lambda)\overline{F_D(\lambda)\lambda^4}d\lambda$$

Eq. S5

where Φ_D is the fluorescence (PL) quantum yield of the donor chromophore, η is the index of refraction of the medium, κ^2 is the dipole-dipole orientation factor, $\sigma_A(\lambda)$ is the wavelength-dependent absorptivity of the acceptor chromophore species, and F_D is the fluorescence emission spectrum normalized by its area. Note that $\int \sigma_A(\lambda)\overline{F_D(\lambda)}\lambda^4 d\lambda$ is referred to as the spectral overlap integral and often denoted as J.

For our CdSe:Te/CdS system, we establish estimates of the various parameters used to determine R_o. We experimentally measured Φ_D = 23% using photothermal deflection, as in Supporting Ref (*46*). We assume the index of refraction η to calculate FRET-based transport to be between 1.8 - 2.0 based on Dement *et. al.*'s investigation of CdSe/CdS QD thin films(*53*).

The last two parameters, the spectral overlap integral and κ^2, are potentially the greatest sources of uncertainty. For κ^2, a conservative estimate is typically to assume that the transition dipole moments of all chromophores in the ensemble are isotropically distributed, such that $\langle\kappa^2\rangle = 2/3$. In a highly ordered superlattice, however, the orientation of QD transition dipole moments could be more aligned, yielding more optimal configurations than 2/3 for $\langle\kappa^2\rangle$. Here, for an estimate of R_o, we use $\langle\kappa^2\rangle = 2/3$. To measure the extinction coefficient of QDs, we measured the absorption spectrum of a stock solution of CdSe:Te/CdS QDs with a 45 mg/mL concentration that is diluted 100-fold. Based on Striolo *et. al.*'s measurements using membrane osmometry as a benchmark for correlating CdSe nanocrystal diameter with molecular weight(*54*), and taking into account the relative amounts of Cd, Se, S, and Te in our system, we crudely estimate the molecular weight of our CdSe:Te/CdS nanocrystals to be 1000 kg/mol. Based on these values and **Eq. S5**, the theoretical R_o is ~ 3.7 nm. This value can be higher with a more generous estimate of extinction coefficient or better aligned transition dipoles between donor and acceptor QDs. Based on the kinetic Monte Carlo simulations described below, we compute that the required R_o to support the measured migration length of 35 nm within the 5 ns TRUSTED measurement window is ~ 15 nm. Because of the $1/r^6$ scaling of FRET, the discrepancy in R_o result in 100-1000 times difference in exciton diffusivity. It is common that FRET theory underestimates the measured exciton transport rate in quantum dot solids(*9*, *11*, *16*, *17*).

6. Kinetic Monte Carlo simulation setup

We used Kinetic Monte Carlo (KMC) simulations to examine whether adjusting R_o alone can reconcile all measurements of energy transfer.

In a hexagonally packed QD grid, each QD is assigned an energy randomly sampled from a distribution corresponding to the peak emission distribution from the single-particle measurements (**Fig. 1D,E**). The FRET rate from a donor QD to every other possible acceptor QD is calculated from Eq. **S4 and S5** above. The spectral overlap component $\int \sigma_A(\lambda)\overline{F_D(\lambda)}\lambda^4 d\lambda$ can be rewritten in terms of energy ε as:

$$\int \frac{\sigma_A(\varepsilon)F_D(\varepsilon)}{\varepsilon^4} d\varepsilon$$

Eq. S6

We used values of Φ_D and η according to **Section 5**. Dipole orientations of all QDs are assumed to have a random orientation, so $\kappa^2 = 2/3$ for all donor and acceptor pairs. The spectral overlap integral is calculated using

$$\sigma_A(\varepsilon) = \sigma_A(\varepsilon_A) \exp\left[-\frac{(\varepsilon - \varepsilon_A)^2}{2\sigma_h^2}\right]$$

Eq. S7

and

$$F_D(\varepsilon) = \frac{1}{\sigma_h\sqrt{2\pi}} \exp\left[-\frac{(\varepsilon - \varepsilon_D + \Delta_{SS})^2}{2\sigma_h^2}\right]$$

Eq. S8

where $\sigma_A(\varepsilon)$ is the acceptor absorption cross section and $F_D(\varepsilon)$ is the donor emission spectrum whose integral is normalized to one. Each QD possesses an intrinsic linewidth of σ_h = 74 meV (174 meV FWHM), the median of the single particle emission FWHM distribution determined by the single particle measurements. ε_A and ε_D are the randomly generated peak absorption energies of the acceptor and donor QDs, according to the distribution in **Fig. 1D,E**, with σ_{ih} = 80 meV. Δ_{ss} represents the Stokes shift.

The energy transfer rate between a donor and an acceptor is determined by **Eq. 2 in the main text.** The time for the transfer event is the inverse of the summation of all rates. An acceptor site for a given hop is selected by a random distribution weighted by the rate of each pair. In other words, pairwise interactions with faster rates are more likely to be chosen.

In the simulations below, we aim to examine each r dependence in **Eq. 2 of main text** separately by matching the measured exciton diffusion extent within the TRUSTED window, 35 ± 6 nm, with simulation and comparing the resulting mean exciton energy relaxation to the experimental TRES. Specifically, we changed the r dependence in the rate equation and adjusted the $\sigma_A(\varepsilon_A)$ as a linear coefficient of energy transfer rate to match the 35 nm exciton diffusion extent, while keeping other parameters untouched. **Fig. S15** shows the resulting decay of mean exciton energy

versus the experimental TRES data. Clearly, r^{-6} dependency produces an energy decay that is too fast while the r^{-2} regime predicts a decay that is too slow. This strongly suggests that there are multiple exciton transport pathways with different r dependencies in QDSL.

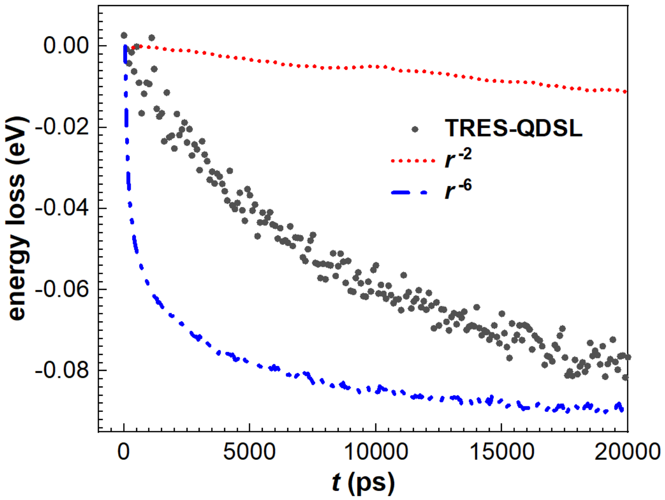

Figure S15. Comparison of far-field and near-field coupling terms in isolation attempting to reconcile the TRUSTED and TRES experimental results.

To evaluate the relative amplitude of the three regimes of dipole-dipole coupling, we used **Eq. 2 of main text** to calculate energy transfer rate in the simulation. We gradually increase c_{far} and adjust $\sigma_A(\varepsilon_A)$ accordingly so that a matching between TRUSTED and TRES may be achieved simultaneously. In the example below, when c_{far}~ 27, both TRUSTED and TRES experimental data can be reproduced by the simulation.

Contribution of near-field and far-field components in the model

As explained in the main text, when the energy transfer rate is described as

$$k = \frac{1}{\tau_D} \frac{R_{o,}^6}{r^6} (1 + c_{\text{intermed}} (2\pi r/\lambda)^2 + c_{\text{far}} (2\pi r/\lambda)^4) \exp(-\alpha r)$$

Eq. S9

with c_{far} ~ 27, and the average R_o = 7.6 nm, both the 35 nm exciton diffusion extent within the TRUSTED window and the energy relaxation dynamics from TRES can be reconciled.

To investigate the contribution of near-field coupling, we performed simulations using strictly near-field interactions with

$$k = \frac{1}{\tau_D} \frac{1}{r^6} (R_o^6) \exp(-\alpha r).$$

Eq. S10

In other words, as a control, the far-field component was removed, and all other parameters were retained. With **Eq. S10** as the energy transfer rate, the simulation yields a 3.7 nm exciton

diffusion extent within the TRUSTED window. Thus, the far-field coupling is essential to increase the exciton diffusion extent to the experimental value of 35 nm.

In addition, we analytically calculated the percentage of near-, intermediate-, and far-field coupling mediated hops. To do this, we multiplied the three terms individually by the number of acceptors, which is proportional to r^2, and performed integration along r out to infinity. This calculation shows that 6% of the rate is contributed by the intermediate- and far-field coupling terms.To identify the relative contributions to c_{far} we note that in the isotropic limit, it includes a factor of 1/3 due to orientational averaging of the projection of donor and acceptor transition dipole moments. While this orientational factor has already been accounted for, it remains to account for the following: we estimate using Gaussian spectral profiles that the far-field overlap integral $\int \sigma_A(\varepsilon) F_D(\varepsilon) d\varepsilon$ is 17× greater than the corresponding weighted integral for the near-field term embodied in R_o in **Eq. S6**. As a result, we find that the relative strength of the far field term requires a remaining factor of 27/17≈5 to adequately model the experimental data. This estimate is only approximate because **Eq. S9** accounts for the scaling with λ (or ε) in the near-field term without the careful integration that is otherwise carried out in **Eq. S6**. The possible contributions to this factor, irrespective of its absolute amount, are discussed in the main text. We also note that the full relative strength of far-field to near-field contributions to the energy transfer rate is smaller than 1 part in 10^6, as one must also account for the factor of $(2\pi r/\lambda)^4$ that we used to non-dimensionalize c_{far}.

7. Electrodynamics simulation demonstrate enhanced far-field over near-field strength

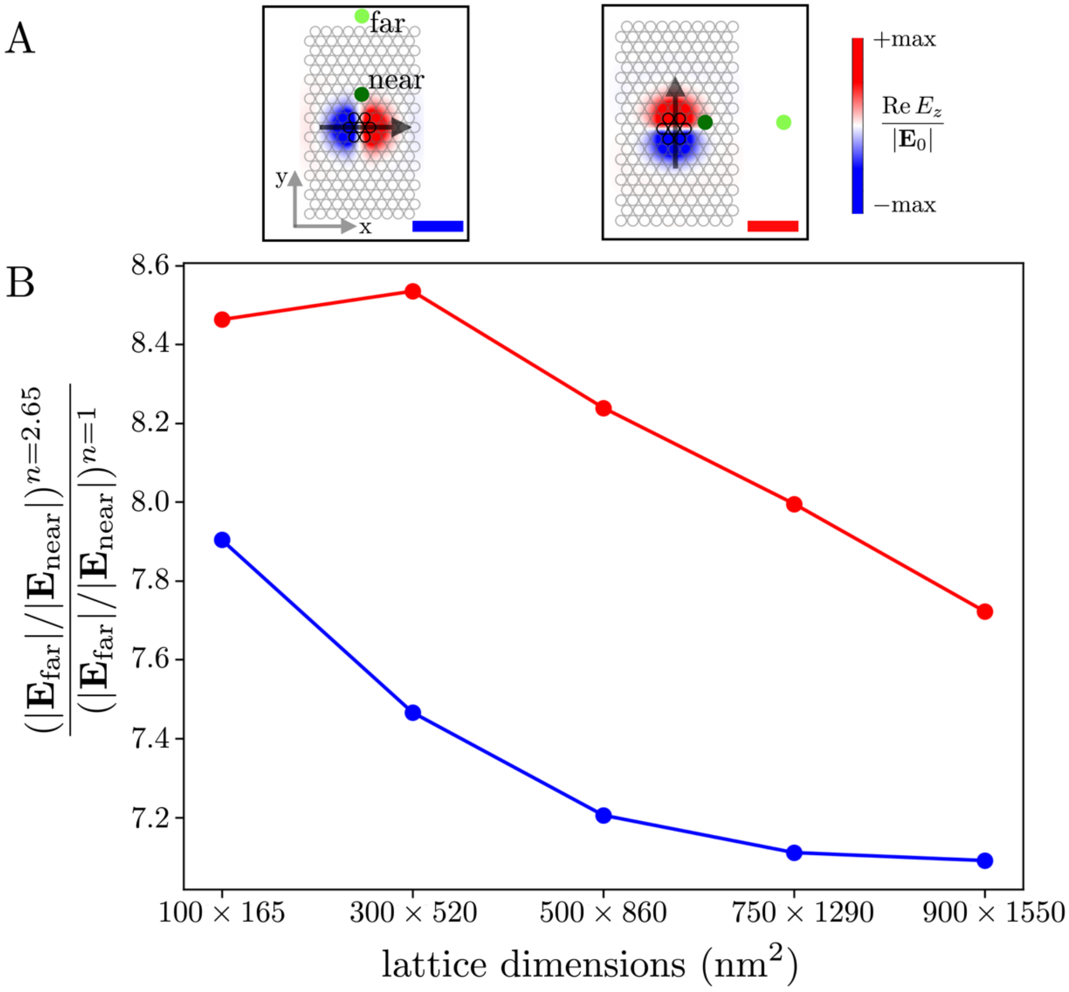

Figure S16: **Comparison of far-field to near-field electric field strength computed in hexagonally packed CdSe QD superlattices with background refractive indices of *n*=2.65 and *n*=1 as a function of overall lattice size.** (A) Representation of the two studied localized QD emission polarization directions and points of observation: (left) horizontal emission polarization and vertical observation, (right) vertical emission polarization and horizontal observation. The out-of-plane component of the localized QD emission electric field (E_z) is displayed in each case. (B) Computed far-field to near-field electric field magnitude ratios corresponding by color to each case in (A) as a function of overall lattice size with background refractive indices of n=2.65 and n=1. In each case the far- and near-field observation points are 7 µm and 30 nm away from the QD excitation region, and the excitation frequency is 2.98×10^{15} rad·sec^{-1}.

Figure S16 illustrates the ratio of the far- to near-field electric field magnitude as a function of hexagonally packed "CdSe QD" superlattice size for background refractive indices of n=2.65 and n=1. Within the coupled dipole approximation(*55–57*), the QD polarization in each lattice point is represented as a point electric dipole that can be polarized by an applied excitation electric field ($\boldsymbol{E}_0$) as well as by the fully retarded electric dipole fields of all surrounding lattice

points. The polarizability of each effective CdSe QD is taken from the *l*=1 electric Mie coefficient corresponding to a 9 nm diameter sphere using the frequency-dependent bulk optical constants from Ref. (*58*). The center-to-center distance between adjacent QD spheres is 10 nm. In each case considered, only a single hexagonal QD cluster positioned at the lattice center is excited at frequency 2.98×10^{15} rad·sec^{-1} by windowing an applied plane wave electric field to that cluster (shown as the black central hexagon in panel A). The QD emission and excitation frequencies and polarizations are the same as the applied field. Two directions are considered for the localized QD excitation/emission polarization (horizontal and vertical) with corresponding in-plane observation points oriented perpendicular to the polarization direction to maximize overlap with the far-field lobes of the excited QD dipoles as shown in panel A. Flooding the background with the CdSe refractive index of n=2.65 (at frequency 2.98×10^{15} rad·sec^{-1}) is chosen in the computed finite lattice systems to better approximate the wavelength contraction effects that would occur in our experimentally studied CdSe QD superlattices. Panel B compares the ratio of the far-field magnitude (computed 7 μm ≈ 10·λ away from the excited QD region) to the near-field magnitude (computed 30 nm ≪ λ away from the excited QD region) in the higher index (n=2.65) medium compared to vacuum (n=1), where λ is the in-medium emission wavelength. A 7-8fold enhancement of the far-field over the near-field is evident across all lattice dimensions studied; similar enhancement would remain even when extrapolating the lattice to the larger dimensions relevant to experiment. This latter effect is already suggested by comparing the left-hand system configuration to the right-hand one in panel A, since on the left there is more polarizable lattice between the source and far-field observation point, and the field ratios in B appear to already asymptote near a value of 7. Taken together, these computational results include both the waveguiding effects occurring in finite CdSe QD superlattices as well as the associated wavelength contraction effects experienced by the electromagnetic signals relaying energy between donor and acceptor points within the lattice, and show a lattice-induced enhancement in the far- to near-field ratio in all cases studied numerically.

8. Far field coupling inclusion into previous literature results

We also performed KMC simulation to test our model on literature results. We chose to test our model on the QD solid measured by Akselrod *et al*(*9*) because they examined the decay of mean exciton energy by TRES as well as spatiotemporal exciton migration via transient PL microscopy. However, no direct connection was established between the two experiments. To do the KMC simulation, we use parameters corresponding to their "d_1" QDs, σ_h = 30 meV, σ_{ih} = 23 meV, Δ_{ss} = 38 meV. Similarly, σ_A is adjusted to match the experimental transient PL microscopy or TRES results. Here, c_{far} ~ 35 is needed to reconcile their reported exciton diffusion length and energy relaxation rate. Furthermore, in this case, R_o becomes ~ 5 nm as opposed to 11 nm obtained with FRET alone,(*9*) well within the reasonable range of estimation based on steady state spectral measurements.(*22*). This example demonstrates that by tuning the inhomogeneity of the QD linewidth, one may control the ratio between near-field and far-field interactions."

9. Tuning the far-field contribution to exciton transport via QD energy inhomogeneity

Based on the Kinetic Monte Carlo simulation that reproduces both the TRUSTED and TRES data of the 5% Te doped CdSe/CdS QDSL (labeled "control simulation"), we repeated the simulation while systematically tuning the intrinsic and inhomogeneous linewidth of the QD absorption/emission spectrum, all the while maintaining the same total linewidth. We report the percentage of exciton hops whose displacement is greater than 40 nm (POH40) over the total number of exciton hops at both 0 ns and 20 ns after photoexcitation.

First, with a FRET-only model, the percentage of hops that exceed 40 nm is extremely small, on the order of 0.1%, as is shown in the first column of **Table S2** labeled "FRET only."

Second, as the intrinsic linewidth is decreased and the inhomogeneous linewidth is increased from columns "Less inhomogeneous" to "Control simulation" to "More inhomogeneous," while POH40 at 0 ns is similar in each case, POH40 differs substantially at 20 ns. For the less inhomogeneous system, POH40 increases to 9.7%, in contrast to the decrease to 4.2% exhibited by the more inhomogeneous system. This difference is in part due to the complex interplay of three energetic parameters that determine energy resonance in the dipole-dipole coupling theory: Stokes shift, inhomogeneous linewidth, and intrinsic linewidth[10]. This example demonstrates that by tuning the inhomogeneity of the QD linewidth, one may control the ratio between near-field and far-field interactions.

Table S2. Tuning QD energy inhomogeneity can alter the effect of far-field interactions.

	FRET only	Less inhomogeneous	Control simulation	More inhomogeneous
Intrinsic linewidth (meV)	74	104.8	74	30
Inhomogeneous linewidth (meV)	80	30	80	104.8
Percentage of far-field hops@0ns	0.13%	5.0%	4.8%	5.6%
Percentage of far-field hops@20ns	0.06%	9.7%	4.8%	4.2%
Diffusion length within 4.8 ns	5.6 nm	46 nm	35 nm	30 nm

REFERENCES AND NOTES

1. Y. Shirasaki, G. J. Supran, M. G. Bawendi, V. Bulović, Emergence of colloidal quantum-dot light-emitting technologies. *Nat. Photonics.* **7**, 13–23 (2013).

2. S. Bisschop, P. Geiregat, T. Aubert, Z. Hens, The impact of core/shell sizes on the optical gain characteristics of CdSe/CdS quantum dots. *ACS Nano* **12**, 9011–9021 (2018).

3. G. H. Carey, A. L. Abdelhady, Z. Ning, S. M. Thon, O. M. Bakr, E. H. Sargent, Colloidal quantum dot solar cells. *Chem. Rev.* **115**, 12732–12763 (2015).

4. M. A. Boles, M. Engel, D. V. Talapin, Self-assembly of colloidal nanocrystals: From intricate structures to functional materials. *Chem. Rev.* **116**, 11220–11289 (2016).

5. C. P. Collier, T. Vossmeyer, J. R. Heath, Nanocrystal superlattices. *Annu. Rev. Phys. Chem.* **49**, 371–404 (1998).

6. C. R. Kagan, E. Lifshitz, E. H. Sargent, D. V. Talapin, Building devices from colloidal quantum dots. *Science* **353**, aac5523 (2016).

7. S. Bellani, A. Bartolotta, A. Agresti, G. Calogero, G. Grancini, A. D. Carlo, E. Kymakis, F. Bonaccorso, Solution-processed two-dimensional materials for next-generation photovoltaics. *Chem. Soc. Rev.* **50**, 11870–11965 (2021).

8. B. Guzelturk, P. L. H. Martinez, Q. Zhang, Q. Xiong, H. Sun, X. W. Sun, A. O. Govorov, H. V. Demir, Excitonics of semiconductor quantum dots and wires for lighting and displays. *Laser Photonics Rev.* **8**, 73–93 (2014).

9. G. M. Akselrod, F. Prins, L. V. Poulikakos, E. M. Y. Lee, M. C. Weidman, A. J. Mork, A. P. Willard, V. Bulović, W. A. Tisdale, Subdiffusive exciton transport in quantum dot solids. *Nano Lett.* **14**, 3556–3562 (2014).

10. F. Prins, A. Sumitro, M. C. Weidman, W. A. Tisdale, Spatially resolved energy transfer in patterned colloidal quantum dot heterostructures. *ACS Appl. Mater. Interfaces* **6**, 3111–3114 (2014).

11. S. J. Yoon, Z. Guo, P. C. dos Santos Claro, E. V. Shevchenko, L. Huang, Direct imaging of long-range exciton transport in quantum dot superlattices by ultrafast microscopy. *ACS Nano* **10**, 7208–7215 (2016).

12. Z. Zhang, J. Sung, D. T. W. Toolan, S. Han, R. Pandya, M. P. Weir, J. Xiao, S. Dowland, M. Liu, A. J. Ryan, R. A. L. Jones, S. Huang, A. Rao, Ultrafast exciton transport at early times in quantum dot solids. *Nat. Mater.* **21**, 533–539 (2022).

13. G. D. Scholes, D. L. Andrews, Resonance energy transfer and quantum dots. *Phys. Rev. B.* **72**, 125331 (2005).

14. N. Kholmicheva, P. Moroz, H. Eckard, G. Jensen, M. Zamkov, Energy transfer in quantum dot solids. *ACS Energy Lett.* **2**, 154–160 (2017).

15. M. S. Azzaro, A. Dodin, D. Y. Zhang, A. P. Willard, S. T. Roberts, Exciton-delocalizing ligands can speed up energy migration in nanocrystal solids. *Nano Lett.* **18**, 3259–3270 (2018).

16. A. J. Mork, M. C. Weidman, F. Prins, W. A. Tisdale, Magnitude of the Förster radius in colloidal quantum dot solids. *J. Phys. Chem. C* **118**, 13920–13928 (2014).

17. K. Zheng, K. Žídek, M. Abdellah, N. Zhu, P. Chábera, N. Lenngren, Q. Chi, T. Pullerits, Directed energy transfer in films of CdSe quantum dots: Beyond the point dipole approximation. *J. Am. Chem. Soc.* **136**, 6259–6268 (2014).

18. D. W. deQuilettes, R. Brenes, M. Laitz, B. T. Motes, M. M. Glazov, V. Bulović, Impact of photon recycling, grain boundaries, and nonlinear recombination on energy transport in semiconductors. *ACS Photonics.* **9**, 110–122 (2022).

19. D. Giovanni, M. Righetto, Q. Zhang, J. W. M. Lim, S. Ramesh, T. C. Sum, Origins of the long-range exciton diffusion in perovskite nanocrystal films: Photon recycling vs exciton hopping. *Light Sci Appl.* **10**, 2 (2021).

20. Z. Zhang, S. J. Kenny, M. Hauser, W. Li, K. Xu, Ultrahigh-throughput single-molecule spectroscopy and spectrally resolved super-resolution microscopy. *Nat. Methods* **12**, 935–938 (2015).

21. R. Yan, S. Moon, S. J. Kenny, K. Xu, Spectrally resolved and functional super-resolution microscopy via ultrahigh-throughput single-molecule spectroscopy. *Acc. Chem. Res.* **51**, 697–705 (2018).

22. S. B. Penwell, L. D. S. Ginsberg, R. Noriega, N. S. Ginsberg, Resolving ultrafast exciton migration in organic solids at the nanoscale. *Nat. Mater.* **16**, 1136–1141 (2017).

23. J. C. Ondry, L. B. Frechette, P. L. Geissler, A. P. Alivisatos, Trade-offs between translational and orientational order in 2D superlattices of polygonal nanocrystals with differing edge count. *Nano Lett.* **22**, 389–395 (2022).

24. A. Avidan, D. Oron, Large blue shift of the biexciton state in tellurium doped CdSe colloidal quantum dots. *Nano Lett.* **8**, 2384–2387 (2008).

25. J. Hanne, H. J. Falk, F. Görlitz, P. Hoyer, J. Engelhardt, S. J. Sahl, S. W. Hell, STED nanoscopy with fluorescent quantum dots. *Nat. Commun.* **6**, 7127 (2015).

26. S. Ye, J. Guo, J. Song, J. Qu, Achieving high-resolution of 21 nm for STED nanoscopy assisted by CdSe@ZnS quantum dots. *Appl. Phys. Lett.* **116**, 041101 (2020).

27. T. Franzl, J. Müller, T. A. Klar, A. L. Rogach, J. Feldmann, D. V. Talapin, H. Weller, CdSe:Te nanocrystals: Band-edge versus Te-related emission. *J. Phys. Chem. C* **111**, 2974–2979 (2007).

28. C. R. Kagan, C. B. Murray, M. Nirmal, M. G. Bawendi, Electronic energy transfer in CdSe quantum dot solids. *Phys. Rev. Lett.* **76**, 1517–1520 (1996).

29. H. Bässler, Charge transport in disordered organic photoconductors a Monte Carlo simulation study. *Phys. Stat. Sol. (b).* **175**, 15–56 (1993).

30. N. Hildebrandt, C. M. Spillmann, W. R. Algar, T. Pons, M. H. Stewart, E. Oh, K. Susumu, S. A. Díaz, J. B. Delehanty, I. L. Medintz, Energy transfer with semiconductor quantum dot bioconjugates: A versatile platform for biosensing, energy harvesting, and other developing applications. *Chem. Rev.* **117**, 536–711 (2017).

31. M. S. Kodaimati, C. Wang, C. Chapman, G. C. Schatz, E. A. Weiss, Distance-dependence of interparticle energy transfer in the near-infrared within electrostatic assemblies of PbS quantum dots. *ACS Nano* **11**, 5041–5050 (2017).

32. P. Geiregat, Y. Justo, S. Abe, S. Flamee, Z. Hens, Giant and broad-band absorption enhancement in colloidal quantum dot monolayers through dipolar coupling. *ACS Nano* **7**, 987–993 (2013).

33. D. L. Andrews, A unified theory of radiative and radiationless molecular energy transfer. *Chem. Phys.* **135**, 195–201 (1989).

34. G. Juzeliūnas, D. L. Andrews, Quantum electrodynamics of resonance energy transfer, in *Advances in Chemical Physics* (John Wiley & Sons Ltd., 2000), pp. 357–410.

35. W. W. Yu, L. Qu, W. Guo, X. Peng, Experimental determination of the extinction coefficient of CdTe, CdSe, and CdS nanocrystals. *Chem. Mater.* **15**, 2854–2860 (2003).

36. A. Bercegol, D. Ory, D. Suchet, S. Cacovich, O. Fournier, J. Rousset, L. Lombez, Quantitative optical assessment of photonic and electronic properties in halide perovskite. *Nat. Commun.* **10**, 1586 (2019).

37. J. P. Dowling, Spontaneous emission in cavities: How much more classical can you get? *Found Phys.* **23**, 895–905 (1993).

38. G. B. dos S. Travassos, thesis, Universidade Federal do Rio de Janeiro, Rio de Janeiro, Brazil (2016).

39. E. M. Purcell, Spontaneous emission probabilities at radio frequencies. *Phys. Rev.* **69**, 681 (1946).

40. M. Janowicz, Decay rate, frequency shift and radiation pattern of a charged harmonic oscillator in the cavity between a metal and a dielectric. *Open Syst Inf Dyn.* **2**, 13–39 (1993).

41. M. Miri, N. Otrooshi, Y. Abdi, Nanoemitter in the vicinity of an impedance plane. *J. Opt. Soc. Am. B, JOSAB.* **30**, 3027–3034 (2013).

42. P. M. de Roque, N. F. van Hulst, R. Sapienza, Nanophotonic boost of intermolecular energy transfer. *New J. Phys.* **17**, 113052 (2015).

43. X. Zhang, C. De-Eknamkul, J. Gu, A. L. Boehmke, V. M. Menon, J. Khurgin, E. Cubukcu, Guiding of visible photons at the ångström thickness limit. *Nat. Nanotechnol.* **14**, 844–850 (2019).

44. J. B. Khurgin, Two-dimensional exciton–polariton—Light guiding by transition metal dichalcogenide monolayers. *Optica.* **2**, 740–742 (2015).

45. C. M. Harris, B. K. Selinger, Single-photon decay spectroscopy. II. The pile-up problem. *Aust. J. Chem.* **32**, 2111–2129 (1979).

46. D. A. Hanifi, N. D. Bronstein, B. A. Koscher, Z. Nett, J. K. Swabeck, K. Takano, A. M. Schwartzberg, L. Maserati, K. Vandewal, Y. van de Burgt, A. Salleo, A. P. Alivisatos, Redefining near-unity luminescence in quantum dots with photothermal threshold quantum yield. *Science* **363**, 1199–1202 (2019).

47. L. Carbone, C. Nobile, M. De Giorgi, F. D. Sala, G. Morello, P. Pompa, M. Hytch, E. Snoeck, A. Fiore, I. R. Franchini, M. Nadasan, A. F. Silvestre, L. Chiodo, S. Kudera, R. Cingolani, R. Krahne, L. Manna, Synthesis and micrometer-scale assembly of colloidal CdSe/CdS nanorods prepared by a seeded growth approach. *Nano Lett.* **7**, 2942–2950 (2007).

48. J. Jasieniak, L. Smith, J. van Embden, P. Mulvaney, M. Califano, Re-examination of the size-dependent absorption properties of CdSe quantum dots. *J. Phys. Chem. C* **113**, 19468–19474 (2009).

49. J. C. Ondry, J. P. Philbin, M. Lostica, E. Rabani, A. P. Alivisatos, Colloidal synthesis path to 2D crystalline quantum dot superlattices. *ACS Nano* **15**, 2251–2262 (2021).

50. A. Dong, J. Chen, P. M. Vora, J. M. Kikkawa, C. B. Murray, Binary nanocrystal superlattice membranes self-assembled at the liquid–air interface. *Nature* **466**, 474–477 (2010).

51. L. Manna, D. J. Milliron, A. Meisel, E. C. Scher, A. P. Alivisatos, Controlled growth of tetrapod-branched inorganic nanocrystals. *Nat. Mater.* **2**, 382–385 (2003).

52. N. D. Bronstein, Y. Yao, L. Xu, E. O'Brien, A. S. Powers, V. E. Ferry, A. P. Alivisatos, R. G. Nuzzo, Quantum dot luminescent concentrator cavity exhibiting 30-fold concentration. *ACS Photonics.* **2**, 1576–1583 (2015).

53. D. B. Dement, M. Puri, V. E. Ferry, Determining the complex refractive index of neat CdSe/CdS quantum dot films. *J. Phys. Chem. C* **122**, 21557–21568 (2018).

54. A. Striolo, J. Ward, J. M. Prausnitz, W. J. Parak, D. Zanchet, D. Gerion, D. Milliron, A. P. Alivisatos, Molecular weight, osmotic second virial coefficient, and extinction coefficient of colloidal CdSe nanocrystals. *J. Phys. Chem. B* **106**, 5500–5505 (2002).

55. M. R. Bourgeois, A. W. Rossi, S. Khorasani, D. J. Masiello, Optical control over thermal distributions in topologically trivial and non-trivial plasmon lattices. *ACS Photonics.* **9**, 3656–3667 (2022).

56. M. R. Bourgeois, A. W. Rossi, M. Chalifour, C. Cherqui, D. J. Masiello, Lattice Kerker effect with plasmonic oligomers. *J. Phys. Chem. C* **125**, 18817–18826 (2021).

57. E. M. Purcell, C. R. Pennypacker, Scattering and absorption of light by nonspherical dielectric grains. *Astrophys. J.* **186**, 705–714 (1973).

58. S. Ninomiya, S. Adachi, Optical properties of cubic and hexagonal CdSe. *J. Appl. Phys.* **78**, 4681–4689 (1995).
